# Supplementary material for: A Protective Role for the Lectin CD169/Siglec-1 against a Pathogenic Murine Retrovirus
Source: Cell Host Microbe. 2019 Jan 9;25(1):87–100.e10. doi: 10.1016/j.chom.2018.11.011 (PMC6331384; doi:10.1016/j.chom.2018.11.011)
Supplement: Document S1. Figures S1–S7 [file mmc1.pdf]

**Cell Host & Microbe, Volume *25***

## Supplemental Information

# A Protective Role for the Lectin CD169/Siglec-1 against a Pathogenic Murine Retrovirus

**Pradeep D. Uchil, Ruoxi Pi, Kelsey A. Haugh, Mark S. Ladinsky, John D. Ventura, Brad S. Barrett, Mario L. Santiago, Pamela J. Bjorkman, George Kassiotis, Xaver Sewald, and Walther Mothes**

**Supplemental Information:**

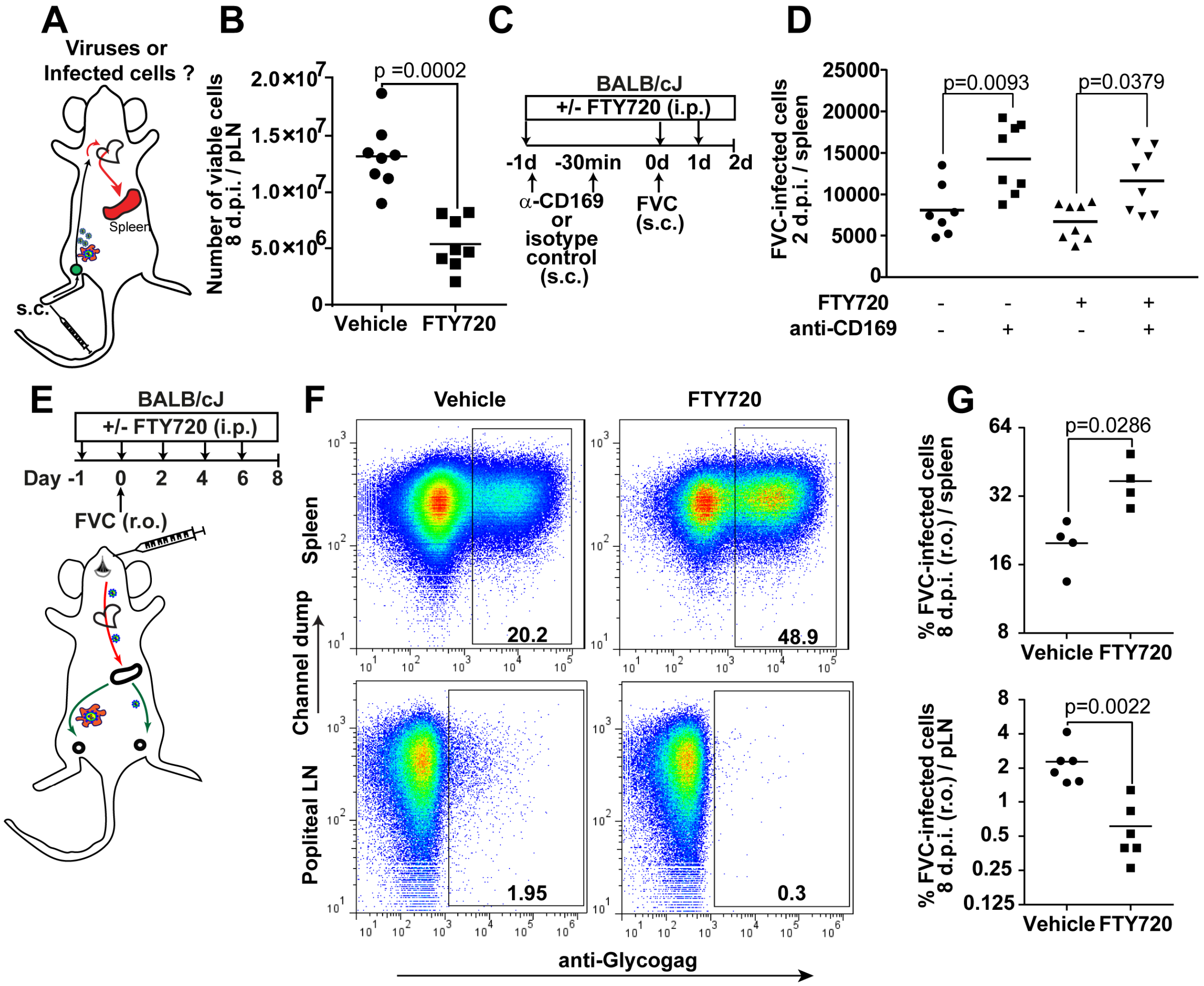


**Figure S1, Related to Figure 2. Retroviruses Use Both Cell-free and Cell-Associated Modes for Dissemination in Mice**

(A) Scheme depicting possible spread of infection via cell-free viruses (blue) or virus-infected cells (orange) from the draining pLN via the subclavian vein (break in the arrow) to the spleen following s.c. administration of virus.

(B) An experiment to show block in lymphocyte infiltration upon FTY720 treatment. After infection, the primary draining lymph node allows the normal entry of lymphocytes from circulation to sample and identify antigen-specific lymphocytes that then proliferate leading to lymph node expansion. FTY720 treatment blocks egress of lymphocytes from all lymphoid

tissue. Thus, fewer lymphocytes enter the circulation as well as infected primary draining lymph node. The graph shows the number of cells in the pLN 8 days after s.c. administration of 2,500 SFFU FVC in BALB/cJ mice (n=8) treated 24 h before and every 24 h for 7 days with vehicle or FTY720. Upon FTY720 treatment the number of cells in the primary draining lymph node did not expand as normal showing efficient block in lymphocyte trafficking.

(C) Flow chart showing route and administration regimen for FTY720, antibodies to CD169 as well as virus.

(D) Number of infected cells in the spleen (n = 7-8) 2 days after s.c. administration of 2,500 SFFU FVC in mice treated with or without antibodies to CD169 or FTY720 as indicated for an experiment shown in C.

(E) Scheme depicting possible modes of spread from the spleen to the pLN after r.o. challenge via cell-free viruses (blue) or virus-infected cells (orange) The flow chart shows the route and administration regimen for FTY720 (i.p.) and FVC in BALB/cJ mice (r.o.; 500 SFFU).

(F) Representative FACS plot showing percentages of FVC-infected (Glycogag$^{+}$) cells after gating on live cells in the spleen and pLN of BALB/cJ mice for an experiment as in E. FTY720 treatment blocks lymphocyte egress leading to their accumulation in the spleen compared to vehicle. There is a concomitant reduction in spread of infection from the spleen to the pLN

(G) Graph showing percentages of FVC-infected cells in the spleen and pLN for an experiment as in E.

Significance p values were obtained using non-parametric Mann-Whitney statistical test.

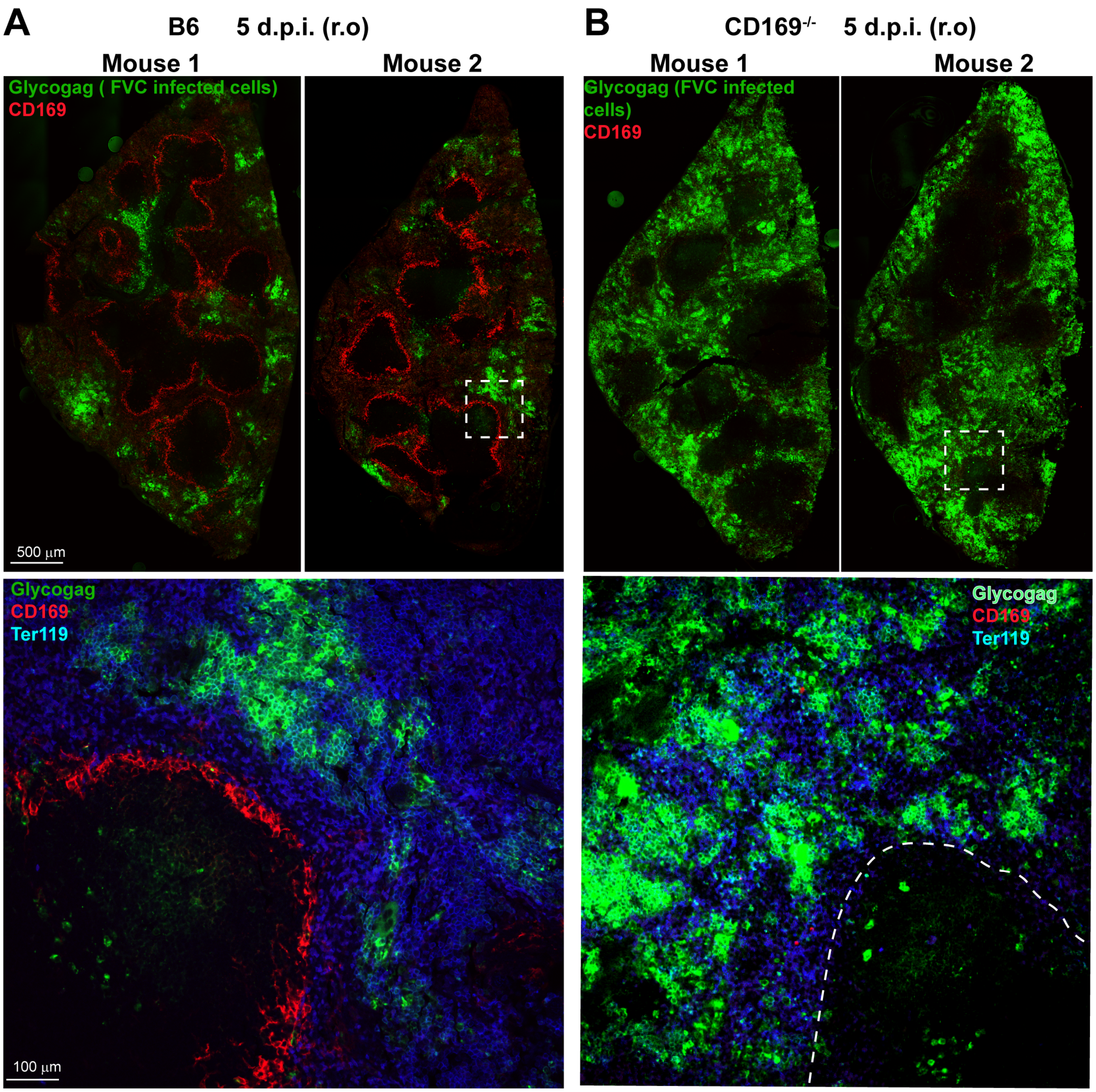


**Figure S2, Related to Figure 4. FVC-infection Spreads Extensively Into the Splenic Red Pulp in CD169$^{-/-}$ Mice**

(A, B) Merged stitched images of immunostained splenic tissue sections are shown for two FVC-infected B6 in A and CD169$^{-/-}$ mice in B (5 d.p.i., r.o., 2,500 SFFU). Metallophilic macrophages within white pulp and FVC-infected cells were identified using antibodies to surface marker CD169 (red) and viral protein Glycogag (green). Magnified images of indicated

insets are shown below that were labeled additionally with antibodies to Ter119 (blue) for staining cells of erythroblast lineages in the red pulp. The red and white pulp border is marked with dotted lines for clarity in B. Scale bars as indicated.

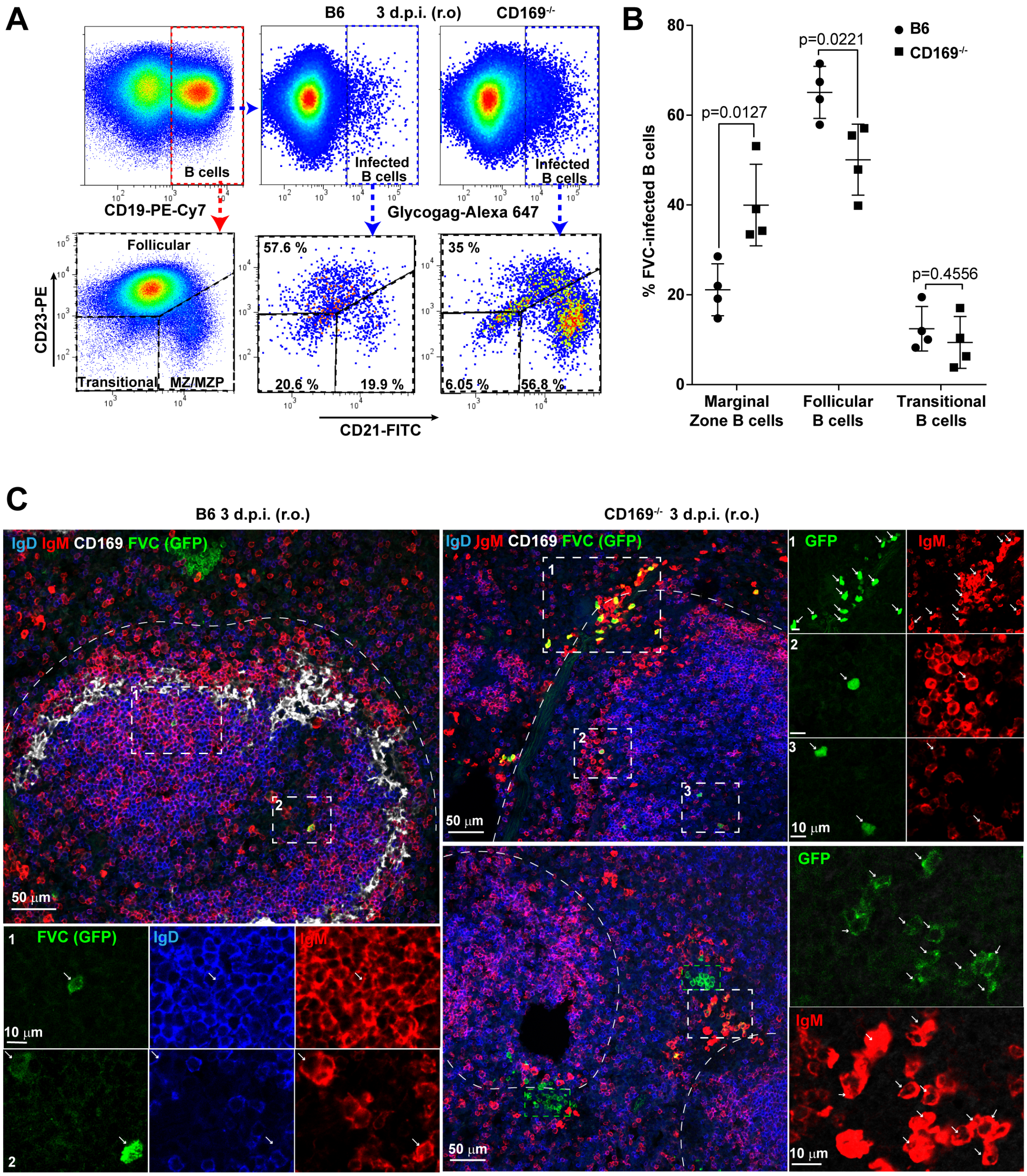


**Figure S3, Related to Figure 4. CD169 Influences the Type of FVC-infected B Cells in the Splenic Marginal Zone**

(A) Gating strategy and representative FACS plot showing characterization of FVC-infected (Glycogag⁺) cells in the CD19⁺ B cell population for presence of mentioned markers in B6 and

CD169$^{-/-}$ mice 3 d.p.i. (n=4, s.c, 2,500 SFFU). The gating strategy used for delineating marginal zone precursor/ marginal zone B (MZ/MZP) cells from follicular (FO) and transitional B cells in the CD23 vs CD21 plot.

(B) Graph showing percentages of MZ/MZP, FO and transitional B cells in the FVC-infected B cell population for experiment as in A.

(C) Merged immunostaining images of splenic tissue sections from B6 and CD169$^{-/-}$ mice infected with FVC-expressing cytoplasmic GFP (3 d.p.i, r.o.;2 x $10^6$ I.U.). MZ B cells (IgM$^{hi}$), FO B cells (IgD$^+$ IgM$^{lo}$) and metallophilic macrophages were identified using antibodies to surface markers IgM (red), IgD (blue), CD169 (white) respectively. Magnified images of individual channels from numerically labeled areas within the inset are shown either below for B6 or towards the right for CD169$^{-/-}$ mice. GFP$^+$ FVC-infected B cells types (FO and MZ) within the follicle in B6 mice are indicated with arrows. Examples of infected B cells in splenic sections of CD169$^{-/-}$ mice that are largely IgM$^{hi}$ near the marginal zone (dotted white lines) as well as in white pulp are also shown with arrows. Green boxes demarcate clusters of infected erythroblasts that are close to the marginal zones and IgM$^{hi}$ MZ B cells. Scale bars as indicated. *p* values; non-parametric Mann-Whitney test.

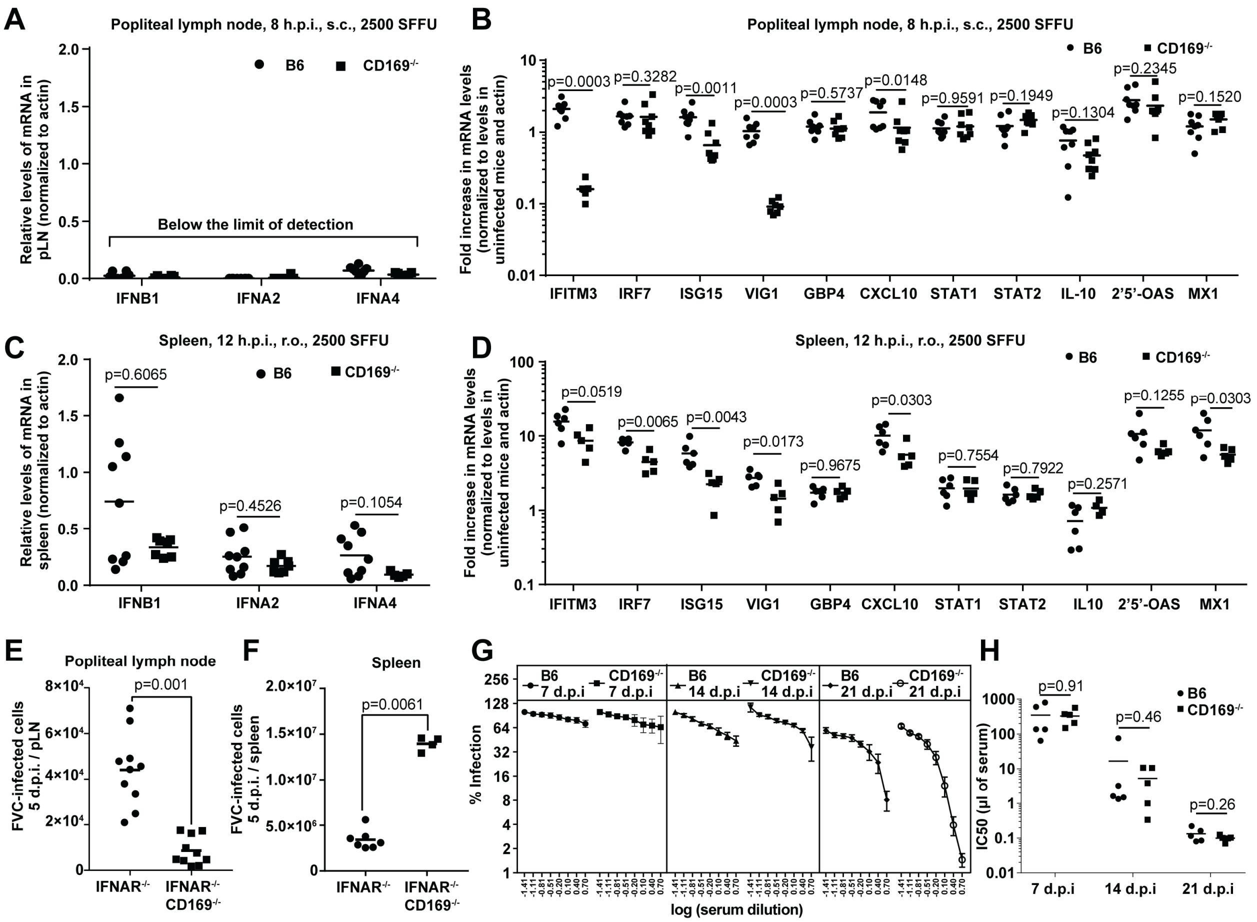


**Figure S4, Related to Figure 6. Innate Immune Response and Neutralizing Antibodies Do Not Contribute to Enhanced Viral Loads in the Spleen of CD169$^{-/-}$ Mice**

(A-D) mRNA levels of indicated interferons (A, C) and interferon-induced genes (B, D) in pLNs (n=6-8) and spleens (n= 4-6) of B6 or CD169$^{-/-}$ mice at specified times and indicated routes of challenge (s.c or r.o) with FVC (2,500 SFFU) as determined by real-time PCR. The mRNA levels were normalized to either actin (A, C) or actin followed by levels in uninfected cells for comparing fold increase (B, D). The levels of interferon RNA were below detection in pLNs under our experimental conditions.

(E, F) FVC-infected cells in the pLN (n = 10) and the spleen (n = 6), 5 d.p.i. after s.c. inoculation with 2,500 SFFU of FVC in *Ifnar1*$^{-/-}$ and *Ifnar1*$^{-/-}$CD169$^{-/-}$ mice.

(G) FVC-neutralizing activity in serially diluted sera from B6 and CD169$^{-/-}$ mice (n=5) at indicated times post subcutaneous challenge with FVC (2,500 SFFU).

(H) Neutralizing titers ($IC_{50}$) in sera from experiment shown in G.

Significance p values were obtained using non-parametric Mann-Whitney statistical test and multiple comparisons were carried out by the Bonferroni-Dunn method using multiple t-test option.

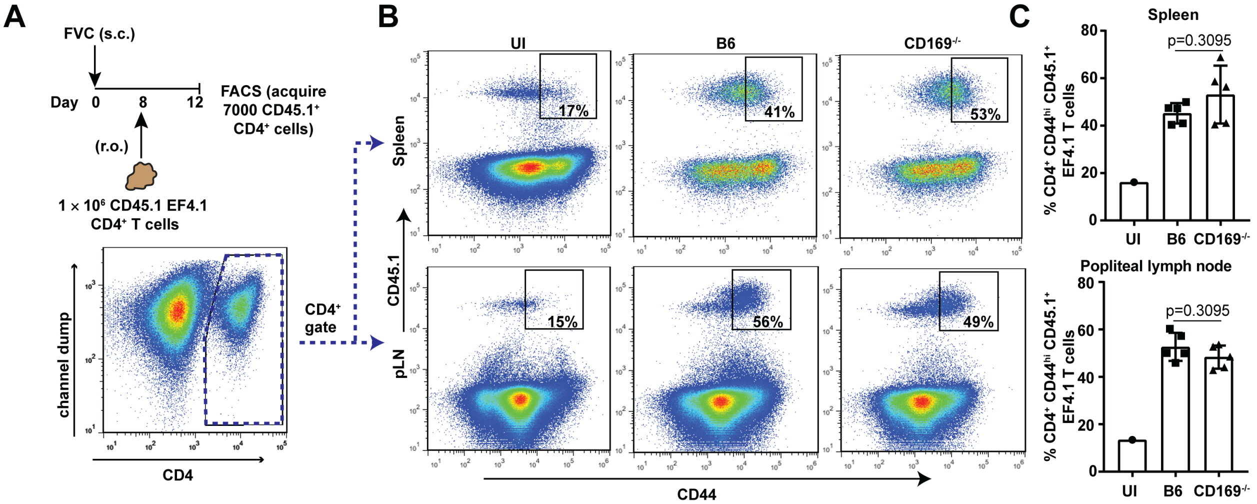


**Figure S5, Related to Figure 6. FVC-Specific CD4$^+$ T Cell Proliferative Responses Are Unaffected in CD169$^{-/-}$ Mice**

(A) Flow chart showing experimental strategy to determine CD4$^+$ T cell proliferation after adoptive transfer of CD45.1 FVC-specific EF4.1 cells in uninfected or FVC-infected B6 or CD169$^{-/-}$ CD45.2 mice (n=5).

(B) Representative FACS plots of splenocytes showing the percentage of CD44$^{hi}$ (proliferating) cells (boxed) among the adoptively transferred CD45.1 EF4.1 cells.

(C) Graph showing percentage of CD44$^{hi}$ proliferating EF4.1 cells at pLN and spleen for experiment shown in B.

Significance p values were obtained using non-parametric Mann-Whitney statistical test.

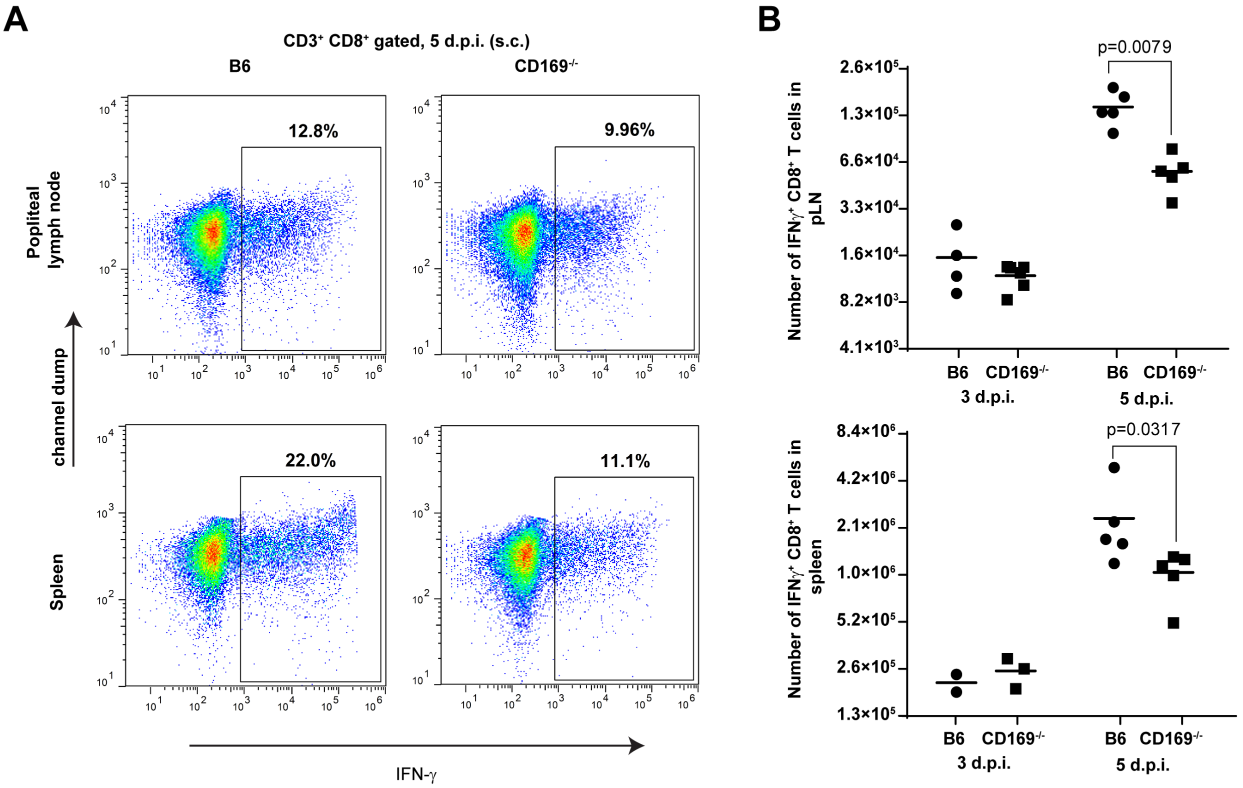


**Figure S6, Related to Figure 6. Induction of IFNγ Producing CD8+ T Cells is Compromised During FVC-Infection in Absence of CD169**

(A) Representative FACS plot for IFNγ+ cells in the CD3+CD8+ gated T cell population at 5 d.p.i. after s.c. delivery of 2,500 SFFU FVC in the draining pLNs (top) and spleens (bottom) from B6 and CD169-/- mice (n=2-5).

(B) Number of IFNγ–producing CD3+CD8+ T cells 3 and 5 d.p.i. for experiment as in A.

Significance p values were obtained using non-parametric Mann-Whitney statistical test.

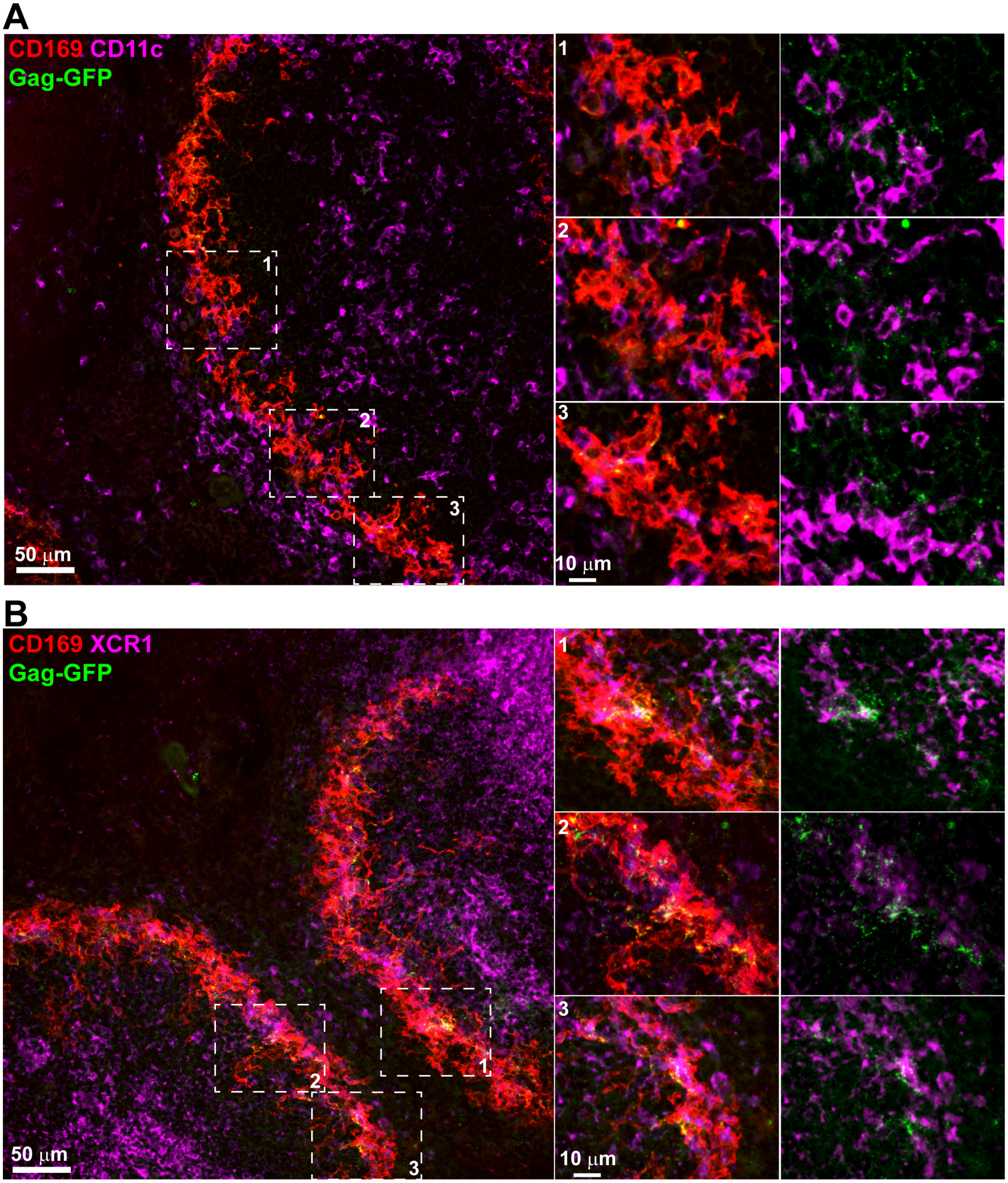


**Figure S7, Related to Figure 7 and Video S2. CD11c$^+$ and XCR1$^+$ DCs Localize in Close Proximity to Retrovirus-Capturing CD169$^+$ Marginal Metallophilic Macrophages**

(A, B) Merged immunostaining images of splenic tissue sections from B6 mice 2 h after r.o. administration (4 x $10^6$ I.U.) of retroviral particles labeled with Gag-GFP (green). DCs, cDC1s and metallophilic macrophages were identified using antibodies to surface markers CD11c (magenta), XCR1 (magenta) and CD169 (red) respectively. Magnified images of numerically labeled areas within the inset are shown towards the right demonstrate close contacts between Gag-GFP labeled virion-capturing $CD169^+$ macrophages and $CD11c^+$ DCs (A) or $XCR1^+$ cDC1s (B).
