# Supplementary material for: A Protective Role for the Lectin CD169/Siglec-1 against a Pathogenic Murine Retrovirus
Source: Cell Host Microbe. 2019 Jan 9;25(1):87–100.e10. doi: 10.1016/j.chom.2018.11.011 (PMC6331384; doi:10.1016/j.chom.2018.11.011)
Supplement: Document S2. Article plus Supplemental Information [file mmc4.pdf]

Article

Cell Host & Microbe

A Protective Role for the Lectin CD169/Siglec-1 against a Pathogenic Murine Retrovirus

Graphical Abstract

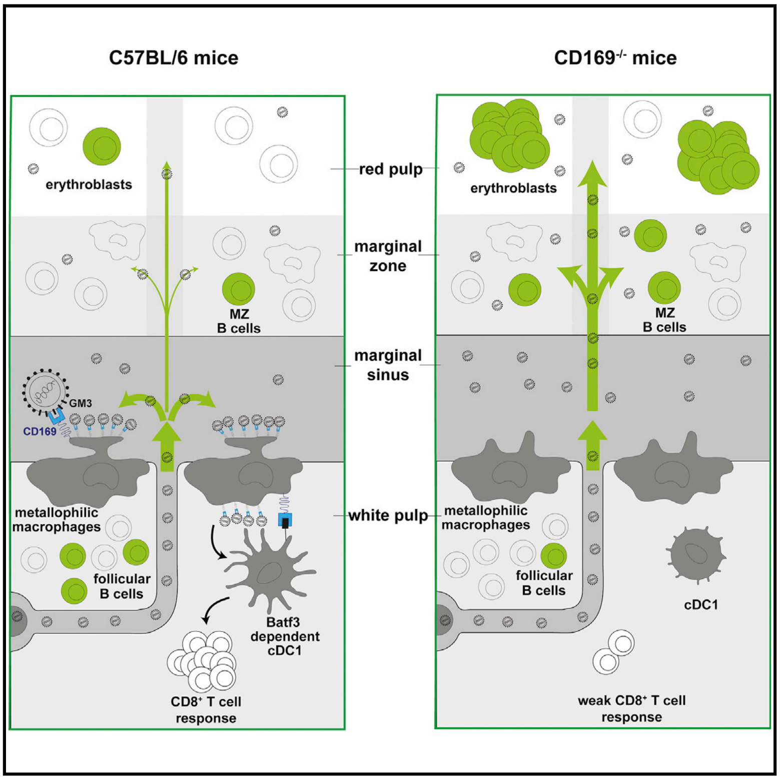

Authors

Pradeep D. Uchil, Ruoxi Pi, Kelsey A. Haugh, ..., George Kassiotis, Xaver Sewald, Walther Mothes

Correspondence

pradeep.uchil@yale.edu (P.D.U.), walther.mothes@yale.edu (W.M.)

In Brief

In a murine model of retrovirus infection, Uchil et al. discover that despite the ability of retroviruses to exploit the surveillance function of sentinel macrophages expressing the lectin CD169 for efficient infection, the immune protective function mediated by CD169 dominates when pathogenic viruses arise with an expanded tropism.

Highlights

- Lectin CD169 limits systemic murine retrovirus dissemination from lymph node to spleen
- CD169 reduces virus spread and infection of erythroblast targets in red pulp of spleen
- CD169 promotes cross-presentation via cDC1 to elicit effective $CD8^+$ T cell responses
- $CD169^{-/-}$ mice have defective antiretroviral $CD8^+$ cytotoxic T cell response

Uchil et al., 2019, Cell Host & Microbe *25*, 87–100
January 9, 2019 © 2018 The Authors. Published by Elsevier Inc.
https://doi.org/10.1016/j.chom.2018.11.011

CellPress

Cell Host & Microbe
Article

A Protective Role for the Lectin CD169/Siglec-1 against a Pathogenic Murine Retrovirus

Pradeep D. Uchil,[1,7,*] Ruoxi Pi,[1,7] Kelsey A. Haugh,[1] Mark S. Ladinsky,[2] John D. Ventura,[1] Brad S. Barrett,[3] Mario L. Santiago,[3] Pamela J. Bjorkman,[2] George Kassiotis,[4] Xaver Sewald,[5,6] and Walther Mothes[1,8,*]

[1]Department of Microbial Pathogenesis, Yale University School of Medicine, New Haven, CT 06510, USA
[2]Department of Biology and Biological Engineering, California Institute of Technology, Pasadena, CA 91125, USA
[3]Division of Infectious Diseases, University of Colorado Denver, 12700 East 19th Avenue, Aurora, CO 80045, USA
[4]Retrovirus Immunology, The Francis Crick Institute, 1 Midland Road, London NW1 1AT, UK
[5]Max von Pettenkofer Institute & Gene Center, Virology, National Reference Center for Retroviruses, Faculty of Medicine, LMU München, Munich, Germany
[6]German Center for Infection Research (DZIF), Partner Site Munich, Munich, Germany
[7]These authors contributed equally
[8]Lead Contact
*Correspondence: pradeep.uchil@yale.edu (P.D.U.), walther.mothes@yale.edu (W.M.)
https://doi.org/10.1016/j.chom.2018.11.011

SUMMARY

Lymph- and blood-borne retroviruses exploit CD169/Siglec-1-mediated capture by subcapsular sinus and marginal zone metallophilic macrophages for *trans*-infection of permissive lymphocytes. However, the impact of CD169-mediated virus capture on retrovirus dissemination and pathogenesis *in vivo* is unknown. In a murine model of the splenomegaly-inducing retrovirus Friend virus complex (FVC) infection, we find that while CD169 promoted draining lymph node infection, it limited systemic spread to the spleen. At the spleen, CD169-expressing macrophages captured incoming blood-borne retroviruses and limited their spread to the erythroblasts in the red pulp where FVC manifests its pathogenesis. CD169-mediated retroviral capture activated conventional dendritic cells 1 (cDC1s) and promoted cytotoxic CD8$^+$ T cell responses, resulting in efficient clearing of FVC-infected cells. Accordingly, CD169 blockade led to higher viral loads and accelerated death in susceptible mouse strains. Thus, CD169 plays a protective role during FVC pathogenesis by reducing viral dissemination to erythroblasts and eliciting an effective cytotoxic T lymphocyte response via cDC1s.

INTRODUCTION

Viruses are immotile but can disseminate within the host either by exploiting the natural flow of body fluids or by using mobile cells. Lymph- and blood-filtering lectin CD169/Siglec-1 expressed on sentinel marginal zone metallophilic macrophages (MMMs) and subcapsular sinus (SCS) macrophages plays a crucial role in capturing retroviral particles such as murine leukemia virus (MLV) and human immunodeficiency virus 1 (HIV-1), thereby promoting the transition of virus dissemination from a cell-free to cell-associated mode (Sewald et al., 2015). CD169 specifically interacted with gangliosides on retrovirus particles to promote their capture. Retrovirus-laden SCS macrophages then *trans*-infected susceptible lymphocytes, which further spread the retroviral infection by formation of virological synapses. Importantly, efficient MLV and HIV-1 infection in mouse models required CD169, suggesting that CD169-mediated *trans*-infection of permissive lymphocytes was exploited by retroviruses. However, the impact of CD169-mediated virus capture and promotion of infection on long-term retrovirus dissemination and pathogenesis remains to be investigated.

This is of particular interest because the sentinel macrophages have been observed to play an important role in immune surveillance by capturing antigens, immune complexes, and tumor-derived vesicles from circulation to orchestrate innate, cell-mediated, and humoral immune responses (Pucci et al., 2016; Saunderson et al., 2014). They also produce type I interferon (IFN) in response to viral infections, activate CD8$^+$ T cells, and cross-present cell-associated viral and tumor antigens to CD8$^+$ T cells (Asano et al., 2011; Backer et al., 2010; Bernhard et al., 2015; Honke et al., 2012; Junt et al., 2007). In addition, they have been observed to transfer captured antigens to *Batf3*-dependent XCR1$^+$ CD8α^+ conventional dendritic cells 1 (cDC1s) in the spleen for cross-presentation to CD8$^+$ T cells. The coordination of the immune activities has been ascribed primarily to the sentinel macrophages, but the specific role of the lectin CD169 in these events and during retrovirus infections remains to be elucidated (Sewald et al., 2015; van Dinther et al., 2018).

To study a possible dual role of CD169 expressed on macrophages in promoting virus infection and/or initiating immune responses against the virus infection, we sought to compare the murine non-pathogenic and pathogenic MLV models. Friend MLV (FrMLV) and Friend virus complex (FVC) are two such commonly used retrovirus models in mice. FrMLV is non-pathogenic in adult mice, as the elicited humoral as well as cell-mediated immune response controls the virus infection (Nowinski, 1976). In contrast, FVC can be pathogenic in sensitive strains of mice. Like most pathogenic MLVs (Rosenberg and Jolicoeur, 1997), FVC consists of a replication-competent helper virus (FrMLV) and a co-packaged pathogenesis-conferring

© 2018 The Authors. Published by Elsevier Inc. This is an open access article under the CC BY license (http://creativecommons.org/licenses/by/4.0/).

replication-defective component. The pathogenic component encodes for a fusion-defective truncated envelope glycoprotein (gp55) from spleen focus-forming virus (SFFV). SFFV gp55 is an agonist of the erythropoietin receptor (EpoR) (Chesebro et al., 1990). Gp55 expression activates EpoR signaling in erythroblasts leading to their proliferation in the spleen, and fomenting infection. Therefore, FVC has an expanded tropism as it can establish infection in erythroblasts in addition to lymphocytes. The erythroblasts are prime targets for FVC-induced pathogenesis as their infection and the subsequent chain of events culminate in splenomegaly (Constantinescu et al., 1998; Li et al., 1990).

Susceptibility to FVC infection depends on the specific mouse strain. While C57BL/6J (B6) mice are resistant, BALB/cJ mice succumb to FVC infection due to uncontrolled splenomegaly (Hasenkrug and Chesebro, 1997; Miyazawa et al., 2008). Susceptibility to FVC-induced splenomegaly is genetically determined by the expression of the Friend virus susceptibility 2 sensitive allele (*Fv2*^{s}) (Lilly, 1970). The *Fv2*^{s} allele encodes the short form of stem cell receptor tyrosine kinase (Sf-Stk) and determines the ability of FVC-infected erythroblasts to proliferate autonomously in response to SFFV gp55 (Persons et al., 1999). In addition, mice carrying major histocompatibility complex (MHC) haplotype H-2^{b} (e.g., B6) allow interrogation of the elicited protective immune response, unlike mice with H-2^{d} (e.g., BALB/cJ) that succumb to severe FVC-instigated disease (Hasenkrug and Chesebro, 1997). B6.*Fv2* mice that carry the *Fv2*$^{s/s}$ allele in the B6 background provide a model to study elicited immune responses as they combine the susceptibility to splenomegaly of *Fv2*^{s} mice with high-recovery phenotype of the resistant mouse strains (Marques et al., 2008).

Here, we study the role of CD169 in retrovirus capture at the popliteal lymph node and its subsequent dissemination to the spleen for the murine non-pathogenic retrovirus FrMLV, and compare it with the pathogenic FVC. Our data revealed that by capturing and promoting infection at the draining popliteal lymph node (pLN), CD169 curtailed retrovirus dissemination systemically into the blood and spleen. In contrast to FrMLV, FVC infection was enhanced in CD169$^{-/-}$ mice at the spleen, as CD169 expressed on MMM was required to diminish FVC spread to the susceptible erythroblast population in the red pulp. In addition to acting as a dissemination-limiting factor, the presence of CD169 on MMM was required for effective cDC1 activation and eliciting a protective cytotoxic CD8^{+} T cell response against FVC. Thus, our data show that CD169 plays a protective role in mitigating FVC pathogenesis, firstly by limiting viral dissemination to protect the erythroblast niche from FVC-induced pathogenesis and secondly by eliciting an effective CD8^{+} cytotoxic T lymphocyte (CTL) response via cDC1 activation to eliminate virus-infected cells.

RESULTS

CD169 Limits Systemic Retrovirus Dissemination

Retroviruses delivered subcutaneously (via footpad) are filtered at the draining pLN by CD169^{+} SCS macrophages. In the absence of CD169, viruses could escape the draining lymph node and disseminate systemically, first through the lymphatics, and then enter the blood through one of the two subclavian veins (Shao et al., 2015) to reach the main blood-filtering lymphoid organ, the spleen. We assessed the extent of retrovirus particle spread 1 hr after subcutaneous (s.c.) injection in B6 and CD169$^{-/-}$ mice using luciferase-encoding FrMLV (Figure 1A). We incubated single-cell suspensions from harvested pLNs, spleens, or plasma with MLV-susceptible DFJ8 cells *in vitro* and measured luciferase activity after 36–48 hr. In B6 mice, the majority of the virus particle-associated luciferase activity was present at the pLN. In contrast, the luciferase activity was 10-fold lower in pLNs of CD169$^{-/-}$ mice (Figures 1B–1D), and concomitantly increased in plasma and spleen, indicating that virus escaped from the pLN into the blood to reach the spleen (Figures 1B–1D). These data show that by capturing retroviruses at the draining pLN, CD169 limits systemic dissemination.

We next monitored levels of FrMLV infection at the pLN and spleen in B6 and CD169$^{-/-}$ mice 5 days post infection (dpi) after s.c. challenge. As expected, FrMLV infection at the pLN was significantly higher in B6 than in CD169$^{-/-}$ mice, as previously observed (Sewald et al., 2015) (Figure 1E). Importantly, despite the early high virus particle load in the spleen, FrMLV infection was significantly lower in CD169$^{-/-}$ than in B6 mice (Figures 1D and 1F). These data indicated that CD169-mediated virus capture was also required at the spleen to promote FrMLV infection. Taken together, our data indicate that the non-pathogenic FrMLV likely evolved to exploit CD169-mediated capture to promote infection of its native host due to its coexistence in the murine host over a million years (Figure 1G) (Yap et al., 2014). This exploitation is not detrimental to the murine host, as the elicited immune response eventually controls the FrMLV infection.

CD169 Plays a Protective Role during a Pathogenic Retrovirus Challenge

We next explored whether CD169-dependent virus capture and infection-promoting activities would be detrimental or protective when the retrovirus infection was pathogenic to the host. We used the FVC retrovirus model for this purpose, as it establishes a pathogenic infection in susceptible strains of mice such as BALB/cJ. Since CD169 knockouts were not available in this background, we blocked CD169 function in BALB/cJ mice by subcutaneously delivering blocking or isotype control antibodies before FVC challenge (Figure 2A) (Sewald et al., 2015). We first monitored mortality to FVC infection with 5-fold differing doses of virus inoculum. If CD169 functions as a retrovirus infection-promoting factor, CD169 blockade would extend survival of treated animals. Unexpectedly, CD169 blockade accelerated mortality in mice compared with controls (Figures 2B and 2C). To gain insight into these results, we evaluated viral loads 8 dpi in the draining pLN and the spleen in BALB/cJ mice (Figure 2D). We also measured the spleen weight to determine the extent of splenomegaly. Consistent with an infection-promoting role for CD169, we observed higher numbers of infected cells in the pLN of control animals compared with those treated with CD169-blocking antibodies (Figure 2E). However, CD169 blockade led to higher plasma viral titers in mice than in the isotype controls (Figure 2F). Moreover, in contrast to FrMLV, where CD169 expression in MMMs was required for efficient infection, FVC-infected cell numbers in the spleen and splenomegaly were higher after CD169 blockade compared with the control (Figures 2G and 2H).

CellPress

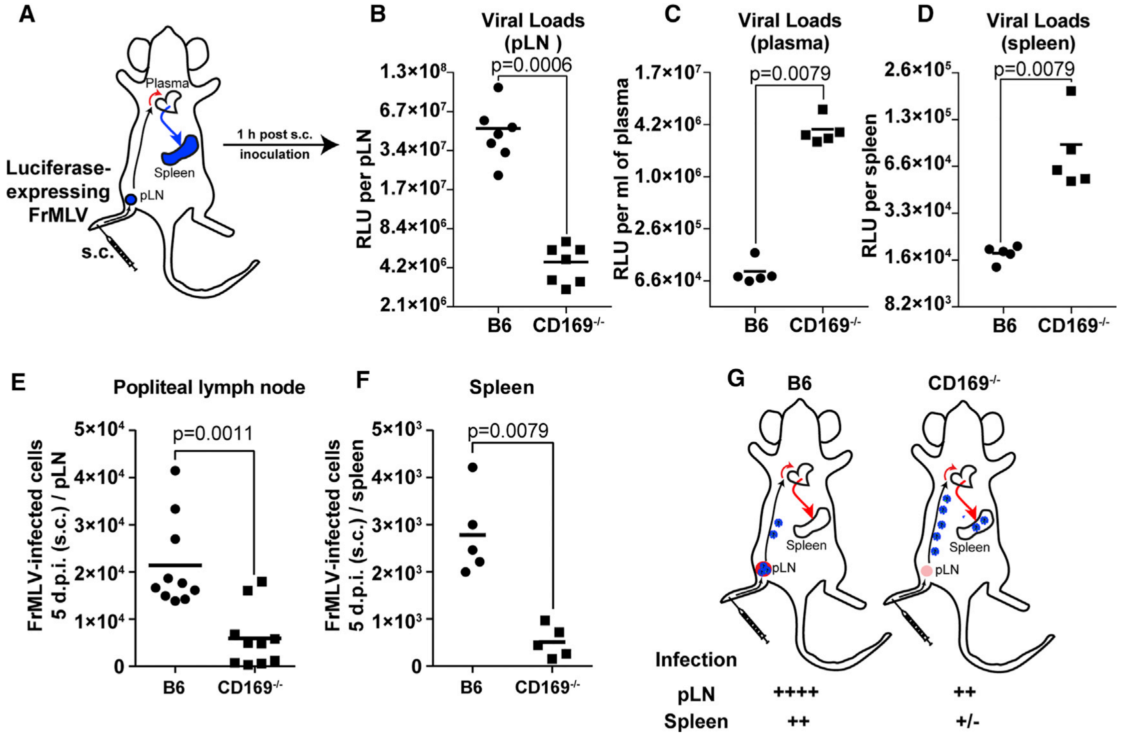

Figure 1. CD169 Limits Retrovirus Dissemination from pLN to Spleen and Is Required for Efficient FrMLV Infection

(A) Scheme indicating a possible path of virus dissemination from popliteal lymph node (pLN) to blood and spleen after subcutaneous (s.c.) footpad administration of luciferase expressing FrMLV.

(B–D) The indicated organs and plasma were harvested 1 hr after virus administration as in (A). The graphs show viral loads measured as relative luciferase units at indicated locations after performing highly sensitive virus load assay in which plasma (n = 5), pLN (n = 7), and splenocyte (n = 5) cell suspensions were incubated with DFJ8 cells for 36–48 hr before measuring luciferase activity.

(E and F) FrMLV-infected cells 5 dpi (s.c., 4×10^5 IU) at pLN (n = 10) and spleen (n = 5) in B6 and CD169$^{-/-}$ mice.

(G) A model depicting FrMLV dissemination and subsequent levels of infection 5 dpi from pLN to blood and the spleen following subcutaneous challenge in B6 and CD169$^{-/-}$ mice to show the infection-promoting role of CD169.

p values derived from non-parametric Mann-Whitney test; mean values denoted by horizontal line.

We tested the potential contribution of cell-free versus cell-associated viruses in spreading infection from the pLN to spleen by treating mice with FTY720 (a potent sphingosine 1-phosphate receptor agonist) to prevent lymphocyte egress from lymphoid tissues (Matloubian et al., 2004) (Figures S1A and S1B). These experiments showed that blocking lymphocyte egress did not influence the enhancement of FVC infection seen in the spleen when CD169 was blocked at the pLN (Figures S1C and S1D). As such, our results corroborated the data obtained for FrMLV spread (Figures 1A–1D), but indicated that in the case of FVC, when CD169 function was compromised, the higher cell-free viral load resulted in enhanced infection at the spleen.

We next challenged both B6 and B6.*Fv2*$^{s/s}$ mice with FVC that, unlike BALB/cJ mice, exhibit a transient splenomegaly, which is heightened in the *Fv2*$^{s/s}$ model before the infection progresses to a low-level chronic phase (Marques et al., 2008; Santiago et al., 2008). As was observed in BALB/cJ mice, the absence of CD169 reduced FVC infection at the draining pLN with concomitant enhancement in the spleen and higher levels of splenomegaly than B6 controls (Figures 2I–2K). B6.*Fv2*$^{s/s}$CD169$^{-/-}$ mice also displayed significantly higher levels of infection in the spleen with heightened splenomegaly than the B6.*Fv2*$^{s/s}$ control mice after s.c. challenge (Figures 2L and 2M). These data corroborated the protective role of CD169 during FVC infection in three mouse models.

In the above experiments, FVC gained entry into the blood via the lymphatics following s.c. administration of the virus. To directly study the role of CD169 for blood-borne retroviruses, we administered FVC via the retro-orbital (r.o.) route. We observed that the number of FVC-infected cells as well as spleen weight were higher in CD169$^{-/-}$ than in B6 mice (Figures 2N and 2P). This indicated enhanced virus replication within the spleen and suggested that CD169 may be required to diminish FVC spread within the tissue architecture of the splenic marginal zone (MZ). We also observed an enhancement in FVC infection at the pLNs of CD169$^{-/-}$ compared with B6 mice (Figure 2Q). Higher systemic viral loads in the absence of CD169 can contribute to increased infection at the pLN (Figures 1B and 2F). In addition, infected cells from the spleen could also be responsible for spreading the infection. We tested this hypothesis by treating mice with FTY720 and monitoring FVC dissemination from the spleen to the pLN at 8 dpi (Figure S1E). Our data revealed that blocking lymphocyte egress led to accumulation of infected cells in the spleen and indeed compromised its dissemination to the pLN (Figures S1E–S1G). These data suggested that both free virus and infected lymphocytes were

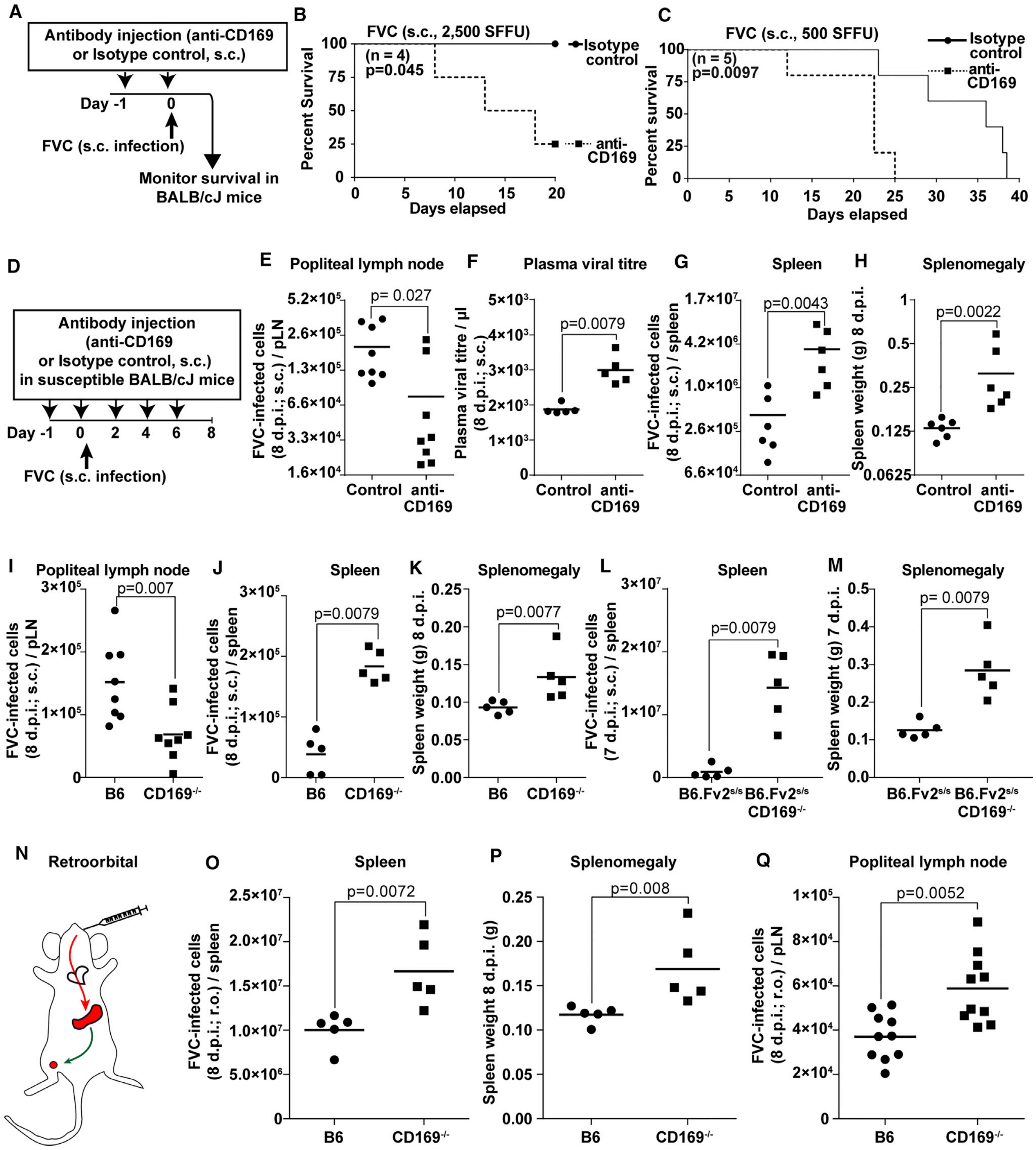

Figure 2. CD169 Plays a Protective Role during Pathogenic FVC Infection

(A–C) Kaplan-Meier survival curves of BALB/cJ mice treated with control or CD169-blocking antibodies (n = 4 or 5 per group) as indicated in the schematic (A) after s.c. challenge with 2,500 spleen focus-forming units (SFFU) (B) or 500 SFFU (C) of FVC.

(D) Scheme showing administration regimen for FVC (s.c. 500 SFFU) and isotype control or CD169 blocking antibody via s.c. injections in BALB/cJ mice over a period of 8 days.

(E–H) FVC-infected cells or plasma virus titer for the experiment outlined in (D) in pLNs (n = 8) (E), plasma (n = 5) (F), and spleen (n = 6) (G), as well as the weight of the spleen (n = 6) (H).

(I–M) FVC-infected cells in the pLN (n = 8) (I) and the spleen (n = 5) (J and L), as well as the weight of the spleen (K and M) at indicated days after s.c. inoculation with 2,500 SFFU of FVC in B6, CD169$^{-/-}$, B6.*Fv2*$^{s/s}$, and B6.*Fv2*$^{s/s}$ CD169$^{-/-}$ mice.

(legend continued on next page)

responsible for spreading infection. Together, our data reveal an unexpected protective role for CD169 against pathogenic FVC in contrast to non-pathogenic FrMLV.

FVC Infection of Erythroblasts Is Enhanced in the Absence of CD169

Blood-borne viruses and antigens are filtered at the spleen in the blood-draining MZ that demarcates the white pulp, and are lined by CD169-expressing macrophages (Martinez-Pomares and Gordon, 2012). In contrast, erythroblasts are located in the red pulp beyond the MZ. Given the ability of FVC to establish infection in the erythroblast population, CD169-mediated retrovirus capture from the blood could diminish virus spread into the red pulp. When CD169 is absent, blood-borne FVC could escape the MZ and gain increased access to erythroblasts in the red pulp for fomenting infection. We tested this hypothesis by measuring the number of FVC-infected cells and erythroblasts ($CD71^+$ $Ter119^+$ $CD19^-$) in the spleen of B6 and $CD169^{-/-}$ mice 5 days after s.c. or r.o. administration (Figures 3A, 3B, 3D, 3F, 3G, and 3I). Strikingly, significantly higher numbers of erythroblasts were infected in the absence of CD169 via both routes. The number of infected B cells was similar (s.c.) or enhanced marginally (r.o.) in $CD169^{-/-}$ mice compared with B6 (Figures 3C and 3H). As a result, the ratios of infected erythroblasts to B cells were significantly enhanced in $CD169^{-/-}$ mice, implying that CD169 may indeed play a protective role by limiting access to erythroblasts in the red pulp (Figures 3E and 3J). Next, we visualized the distribution of FVC-infected cells by immunostaining tissue sections of spleen from B6 and $CD169^{-/-}$ mice 5 dpi (s.c and r.o.). As expected, FVC-infected cells in the spleens of B6 mice after s.c. infection were rare compared with $CD169^{-/-}$ mice due to the virus filtering at the pLN (Figures 4A and 4B). We observed FVC-infected B cells in close vicinity to the $CD169^+$ MMMs at the marginal/follicular zones and minimal erythroblast infection. In contrast, FVC-infected proliferating erythroblasts were clearly visible in the red pulp of $CD169^{-/-}$ mice, suggesting increased virus escape into the red pulp. In splenic sections of r.o. challenged B6 mice, we observed only fewer foci of FVC-infected erythroblasts (Figure 4C). In comparison, FVC-infected erythroblasts occupied most of the red pulp in splenic sections of $CD169^{-/-}$ mice indicating enhanced viral spread in the absence of CD169, corroborating the data obtained after s.c. challenge (Figures 4B and 4C). We were also able to confirm viruses budding out from a cluster of proliferating erythroblasts in the red pulp of splenic sections from $CD169^{-/-}$ mice (5 dpi, r.o.) using electron tomography (Figure 4D; Video S1). Taken together, our data indicated that CD169 expression on MZ macrophages impeded retrovirus dissemination into the red pulp and diminished FVC-induced pathogenesis by protecting the highly susceptible erythroblast niche.

Since CD169 plays a major role in *trans*-infection of underlying B cells at the pLN (Sewald et al., 2015), we also explored whether the absence of CD169 led to changes in infection of specific B cell subtypes in the splenic MZ during FVC infection. CD169-expressing MMM demarcate the white pulp that contains follicular B cells on the inner side and MZ B cells on the outer rim (Arnon et al., 2013; Cerutti et al., 2013; Martin and Kearney, 2002). We characterized FVC-infected B cells into follicular (FO), MZ, and transitional B cells using CD21 and CD23 staining 3 dpi (r.o.) (Meyer-Bahlburg et al., 2008; Oliver et al., 1997) (Figure S3A). Although FO B cells remained the major B cell types targeted by FVC, there was a significant decrease in their infection when CD169 was absent. Consequently, $CD169^{-/-}$ mice showed higher infection of MZ B cells compared with B6 mice (Figure S3B). The percentages of FVC-infected transitional B cells were similar in both groups (Figure S3B). We were also able to visualize infected MZ B cells (high immunoglobulin M [IgM^{hi}]) located near MZs in splenic sections of $CD169^{-/-}$ mice (Figure S3C). Infected FO (IgD^+ IgM^{lo}) and MZ (IgM^{hi} IgD^{lo}) B cells were primarily located within the white pulp of splenic sections in B6 mice (Figure S3C) (Zouali and Richard, 2011). Immunostaining also revealed close proximity of IgM^{hi} MZ B cells to clusters of infected erythroblasts in the red pulp. These data suggested that enhanced infection of erythroblasts, in addition to increased virus flow through the outer MZ, contributed to higher infection of MZ B cells in $CD169^{-/-}$ mice. Thus, in addition to reducing dissemination to the red pulp, CD169 expression influenced the transmission of captured viruses to the target lymphocytes.

Site-Specific Antibody Blockade Reveals the Importance of CD169 at Both the Draining Popliteal Lymph Node and the Spleen

Retroviruses entering the lymph are first captured at the lymph node in the subcapsular sinus, and those that escape into the blood are captured at the splenic MZ by $CD169^+$ macrophages. Hence, there is a dual effect of CD169 on the total level of infection at the spleen. We decided to ascertain the relative importance of these two capture events by impairing CD169 function in a site-specific manner at the pLN and/or spleen using locally administered CD169 blocking antibodies in BALB/cJ mice. We first determined that 5 μg of CD169 antibodies, when delivered subcutaneously, led to its blockade in a site-specific manner at the pLN, but not at the spleen (Figure 5A). We ascertained this by determining the percentages of $CD169^+$ cells in both compartments in comparison with isotype control-treated mice. Similarly, 20 μg of blocking antibody delivered r.o. blocked CD169 in a site-specific manner at the spleen, but not the pLN (Figure 5A). We then challenged mice subcutaneously with FVC when CD169 was blocked at the pLN, spleen, or both organs, and determined the levels of infected cells in the spleen 3 dpi by co-culturing 2×10^6 splenocytes with MLV-susceptible DFJ8 cells to enhance sensitivity. The data reveal that blocking CD169 at the pLN led to a 15-fold increase in FVC infection at the spleen as compared with the isotype control. Blocking CD169 at the spleen alone resulted in a $\sim$5-fold increase in splenic infection, whereas a blockade at both pLN and the

(N) Scheme depicting possible path of blood-borne retrovirus via the heart to spleen, the main blood-filtering lymphoid tissue, and its subsequent spread to secondary draining sites such as pLN following r.o. inoculation.

(O–Q) FVC-infected cells in the spleen (n = 5) (O) and pLN (n = 10) (Q), as well as spleen weight (P), are shown for B6 and $CD169^{-/-}$ mice 8 days after r.o. administration with 2,500 SFFU of FVC.

p values derived from non-parametric Mann-Whitney test; mean values denoted by horizontal line. See also Figure S1.

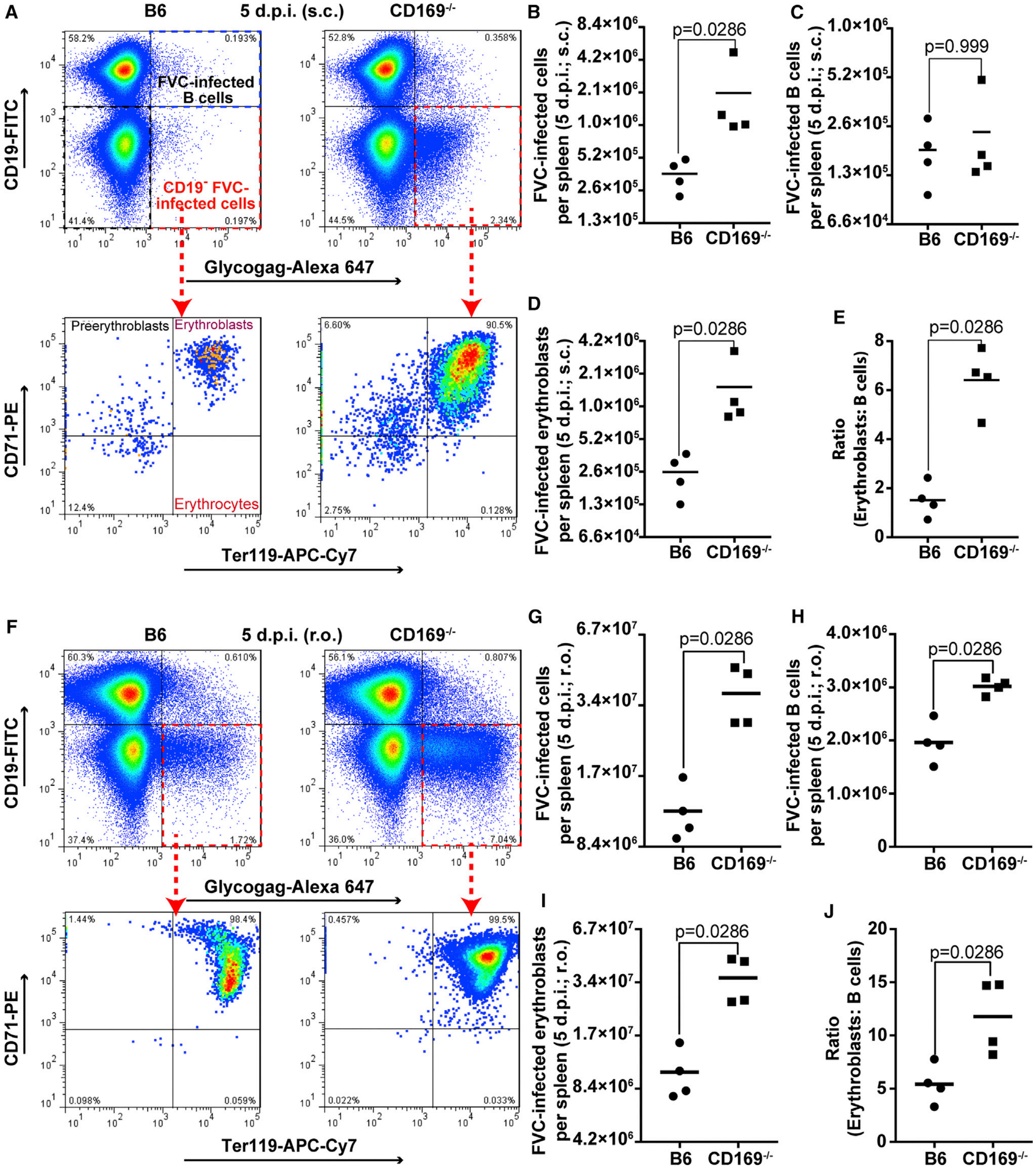

Figure 3. CD169 Reduces FVC Spread to Erythroblasts in the Red Pulp

Fluorescence-activated cell sorting (FACS) plots showing the gating strategy and graphs depicting the numbers of FVC-infected cells, erythroblasts, B cells, and ratios of infected erythroblasts and B cells in splenocytes of B6 and $CD169^{-/-}$ mice (n = 4) 5 days after s.c. (A–E) or r.o. (F–J) administration (2,500 SFFU). Erythroblasts ($CD71^{+}$ $Ter119^{+}$ $CD19^{-}$), B cells ($CD19^{+}$), and FVC-infected cells ($Glycogag^{+}$) were identified using the indicated fluorophore conjugates. p values derived from non-parametric Mann-Whitney test; mean values denoted by horizontal line.

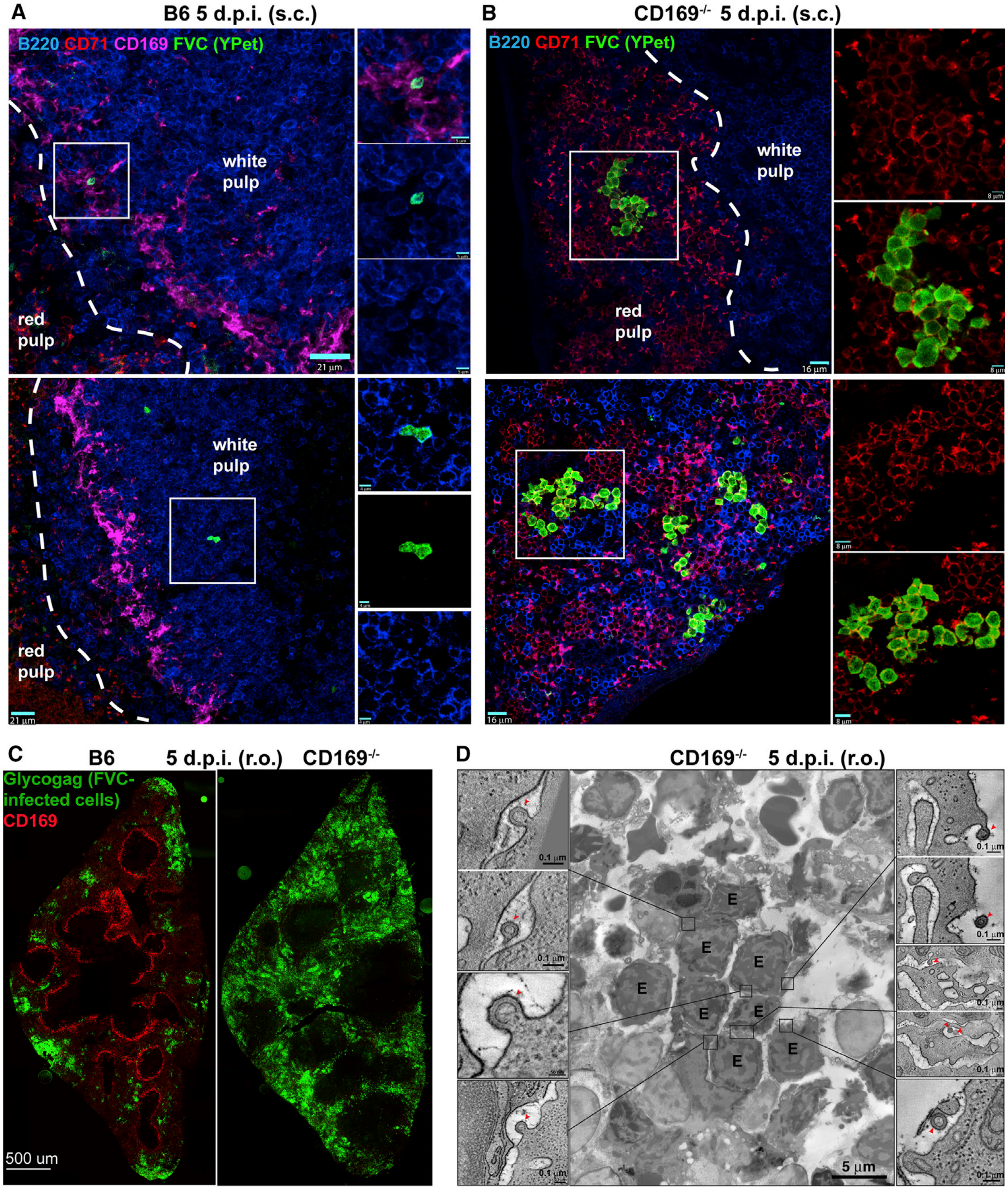

Figure 4. CD169 Limits Dissemination of FVC into Erythroblast Niche of the Splenic Red Pulp

(A and B) Merged immunostaining images of splenic tissue sections from B6 and CD169$^{-/-}$ mice 5 dpi after s.c. administration (2×10^6 IU) of Ypet expressing FVC (green). B cells, erythroblasts, and metallophilic macrophages were identified using antibodies to surface markers B220 (blue), CD71 (red), and CD169 (pink), respectively. The B cell follicular area (white pulp) and extrafollicular erythroblast rich areas (red pulp) are demarcated by dashed white lines. Magnified images of merged and individual channels of insets are shown on the right.

(C) Merged immunostaining images of splenic tissue sections from B6 and CD169$^{-/-}$ mice 5 dpi after r.o. administration of FVC (2,500 SFFU). Metallophilic macrophages lining the white pulp and FVC-infected cells were identified using antibodies to surface marker CD169 (red) and viral protein Glycogag (green).

(D) Electron tomography of a spleen section from CD169$^{-/-}$ mice for an experiment as in (A). The image shows a cluster of clonally expanded FVC-infected erythroblasts (labeled E). Insets show details from serial tomographic reconstructions, demonstrating nascent viruses (red arrowheads) budding from the surfaces or invaginations of infected erythroblasts.

See also Figures S2 and S3 and Video S1. Scale bars as indicated.

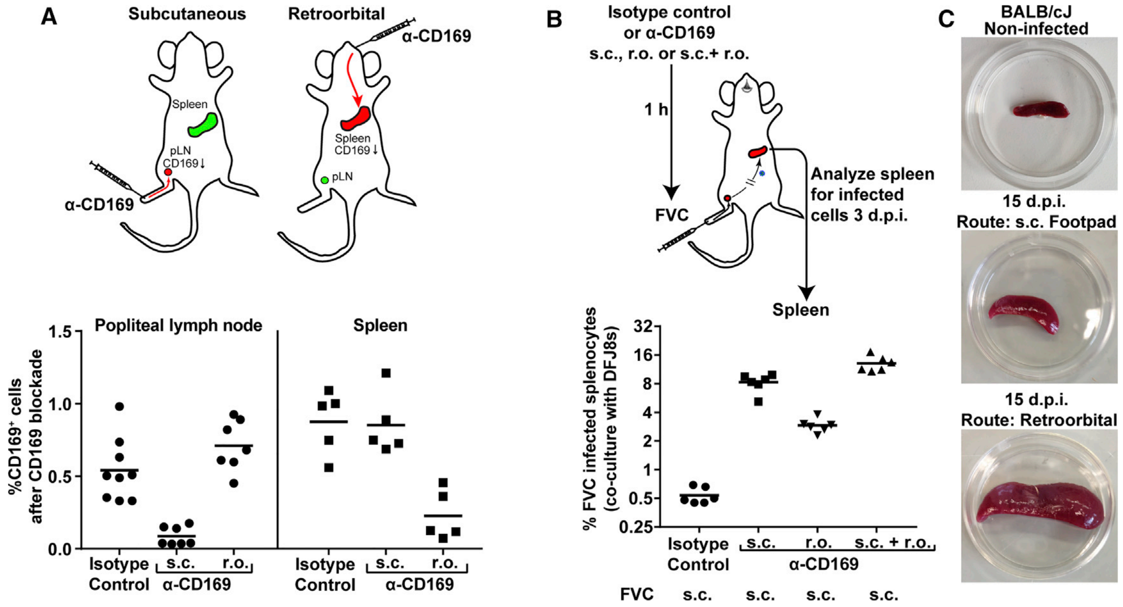

Figure 5. CD169 Function Is Required at Both pLN and Spleen for Limiting Retrovirus Dissemination

(A) The upper panel depicts a scheme showing administration of isotype control or CD169 blocking antibodies via s.c. (5 μg) or r.o. (20 μg) route to elicit site-specific blocking at pLN (n = 7–9) or spleen (n = 5), respectively, in BALB/cJ mice. The graph in the lower panel shows percentages of CD169-positive cells in pLN or spleen, 1 hr after CD169 blockade via indicated routes.

(B) Scheme showing administration of CD169 blocking antibodies via mentioned routes as in (A) followed by s.c. inoculation of BALB/cJ mice (n = 6; 2,500 SFFU) with FVC after 1 hr. The splenocytes were harvested 3 dpi and co-cultured with DFJ8 cells to determine the levels of infection by FACS analyses of Glycogag$^+$ cells.

(C) Image of spleens from BALB/cJ mice that were uninfected or infected with FVC (500 SFFU) 15 dpi via mentioned routes.

p values derived from non-parametric Mann-Whitney test; mean values denoted by horizontal line.

spleen had a synergistic effect with infection levels reaching 24-fold above control antibody-treated animals (Figure 5B). These data suggest that CD169 function is likely important at both the pLN and spleen. However, the contribution of virus-filtering activity at the pLN is higher in the outcome of total infection at the spleen for subcutaneously administered virus. To illustrate this point, we administered equal amounts of virus via the s.c. and r.o. route in BALB/cJ mice and monitored splenomegaly 15 dpi. The images reveal that the extent of infection in the spleen, as indicated by splenomegaly, was drastically low when FVC was administered via the s.c. compared with r.o. route due to the virus-filtering activity of the pLN (Figure 5C).

Analyses of Innate, Humoral, and Cell-Mediated Immune Responses Reveal a Role for CD169 in Eliciting Effective Cytotoxic CD8$^+$ T Cell Activity

In addition to altered dissemination of virus, a blunted innate, humoral, and/or cell-mediated immune response may contribute to the high viral loads observed in CD169$^{-/-}$ mice. Immune response to FVC infection is well characterized (Hasenkrug and Chesebro, 1997). The innate, humoral, and cell-mediated arms of the immune response control FVC during various phases of infection (Hasenkrug and Dittmer, 2000). Given that CD169 macrophages at the pLN are known to produce type I IFNs that can protect neurons from lethal vesicular stomatitis virus (VSV) infection (Iannacone et al., 2010), we assessed the mRNA levels of IFN-α and IFN-β in addition to IFN-stimulated genes (ISGs) in the two groups. Although there were differences in induction of some ISGs (*IFITM3*, *IRF3*, *ISG15*, *VIG1*, and *MX1*), mRNA levels of type I IFNs were below the detection limit in pLNs or similar in spleens of B6 and CD169$^{-/-}$ mice post-FVC infection (Figures S4A–S4D). We functionally tested the role of type I IFNs by generating CD169$^{-/-}$*Ifnar1*$^{-/-}$ mice and found that the infection levels in their spleens were still significantly elevated (5-fold) compared with the control *Ifnar1*$^{-/-}$ mice (Figures S4E and S4F). Thus, the role of CD169 was not rescued in *Ifnar1*$^{-/-}$ mice.

We next assessed the humoral immune response by comparing FVC-neutralizing antibody titers in the sera of B6 and CD169$^{-/-}$ mice at 7, 14, and 21 dpi (s.c.). Neutralizing activity and the calculated IC_{50} in the sera of both groups of mice were similar (Figures S4G and S4H). To compare FVC-specific CD4$^+$ T cell responses in B6 and CD169$^{-/-}$ mice, we utilized EF4.1 mice, which transgenically express a T cell receptor β chain that can specifically recognize MHC class II-presented FrMLV envelope epitope (Antunes et al., 2008). We estimated the percentage of CD44^{hi} proliferating EF4.1 CD4$^+$ T cells (CD45.1) after adoptively transferring them (r.o.) to mice 8 dpi (s.c.) (Figure S5A). Our data suggested that FVC-specific CD4$^+$ T cells proliferated to similar extent in B6 and CD169$^{-/-}$ mice compared with uninfected controls at both spleen and pLN (Figures S5B and S5C). Taken together, these data suggested that type I IFN, humoral, and CD4$^+$ T cell proliferative responses were similar in both groups and excluded their contribution to enhanced viral loads in CD169$^{-/-}$ mice.

The role of MZ CD169$^+$ macrophages in cross-presenting antigens to both CD8$^+$ T cells and DCs is well established

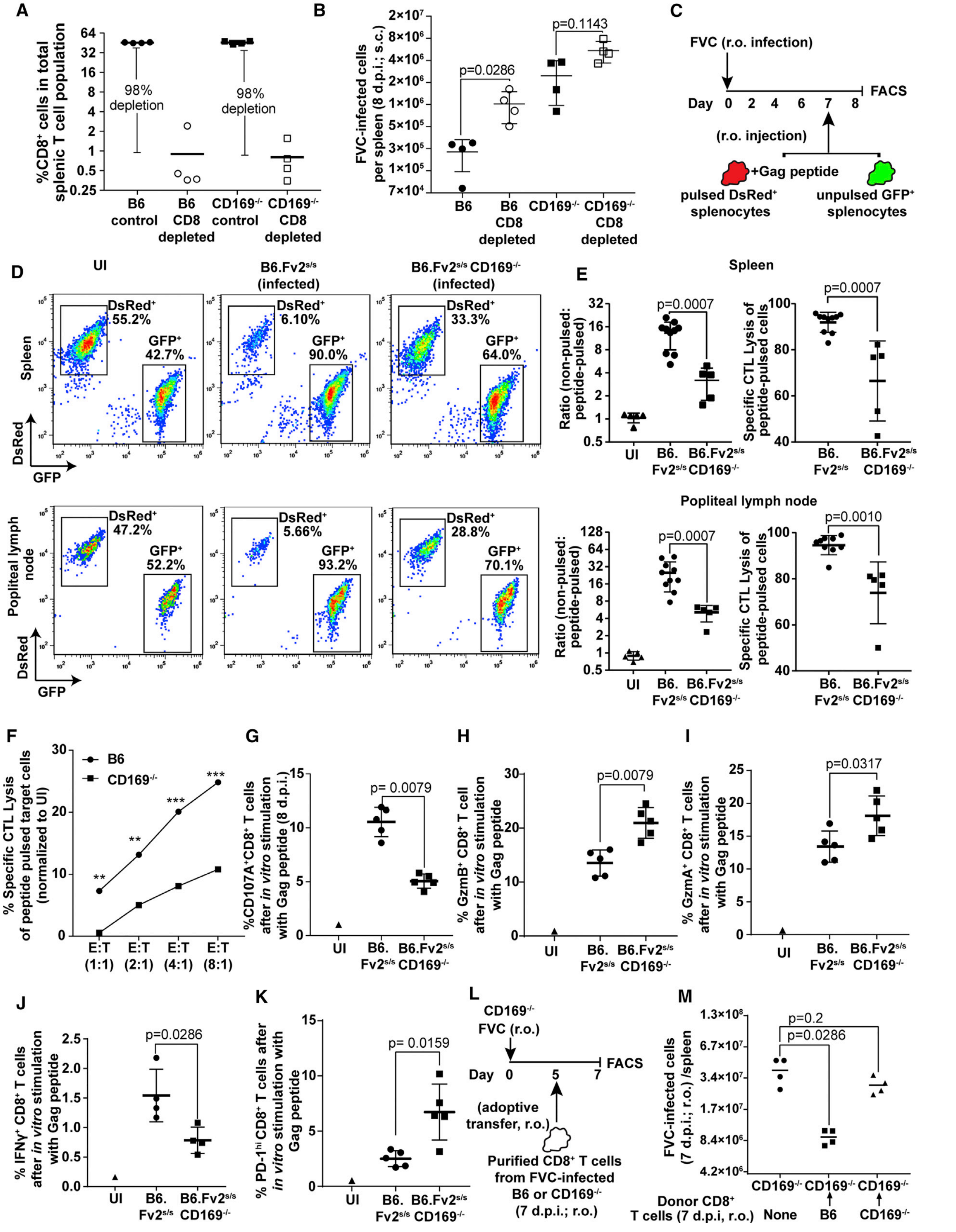

(legend on next page)

(Backer et al., 2010; Bernhard et al., 2015). Therefore, reduced killing of infected cells by impaired CD8$^+$ T cell activity could contribute to the enhanced infection in the absence of CD169. To test this possibility, we first depleted CD8$^+$ T cells using CD8α-specific antibodies in B6 and CD169$^{-/-}$ mice challenged subcutaneously with FVC and monitored infection in the spleen (Figure 6A). There was a significant increase in the infection levels of splenocytes when CD8$^+$ T cells were depleted in B6, but not in CD169$^{-/-}$, mice (Figure 6B). These data suggest that CD8$^+$ T cell activity could be impaired in CD169$^{-/-}$ mice. To test their function directly, we investigated *in vivo* CD8$^+$ CTL activity in the *Fv2*$^{s/s}$ background mice after r.o. challenge with FVC. We adoptively transferred a 1:1 mix of non-pulsed splenocytes from GFP-expressing mice and FrMLV Gag peptide (6 μM) pulsed splenocytes from dsRed-expressing mice 7 dpi (Figure 6C). The ratio of DsRed- and GFP-positive cells were analyzed a day later in the spleen and pLN. The data showed that the FVC-specific CD8$^+$ CTL lysis was significantly impaired in absence of CD169 at both the spleen and pLN (Figures 6D and 6E). We then tested CTL function *in vitro*, using purified CD8$^+$ T cells from B6 and CD169$^{-/-}$ mice 7 dpi and incubating them with peptide-pulsed dsRed and non-pulsed GFP-expressing splenocytes at various effector and target ratios. We observed a diminished ability of CD8$^+$ T cells from CD169$^{-/-}$ mice compared with B6 mice to kill target cells across various effector-to-target ratios tested (Figure 6F). Analyses of degranulation activity by staining for surface exposure of lysosomal marker CD107A following *in vitro* stimulation of infected splenocytes with FrMLV Gag peptide revealed that CD8$^+$ T cells were significantly compromised in their degranulation activity when CD169 was absent (Figure 6G). This led to concomitant intracellular accumulation of cytotoxic granular components (granzyme A and B) (Figures 6H and 6I). Importantly, there was a significant reduction in IFN-γ-producing CD8$^+$ T cells in Gag-peptide- and PMA/ionomycin-stimulated splenocyte culture from CD169$^{-/-}$ mice compared with B6 mice in the Fv2$^{s/s}$ background (Figures 6J and S6). Furthermore, when CD169 was absent, CD8$^+$ T cells showed signs of dysfunction, as they expressed higher levels of the immune checkpoint protein PD-1 (Figure 6K). Finally, we carried out adoptive transfer of primed CD8$^+$ T cells from infected B6 or CD169$^{-/-}$ mice to infected CD169$^{-/-}$ mice (Figure 6L). CD169$^{-/-}$ mice that did not receive CD8$^+$ T cells served as controls. The data revealed the significantly superior ability of CD8$^+$ T cells from B6 compared with CD169$^{-/-}$ mice in reducing FVC-infected cell numbers (Figure 6M). These data complemented the CD8 depletion experiment (Figures 6B and 7C) and indicated that reduced CD8$^+$ CTL activity contributed to enhanced viral loads in addition to altered virus dissemination in CD169$^{-/-}$ mice.

CD169 Contributes to Activation of *Batf3*-Dependent cDC1s for Cross-Priming CD8$^+$ T Cells

In addition to directly priming some CTLs, MMMs also activate *Batf3*-dependent XCR1$^+$ CD8α$^+$ cDC1s by binding to surface sialic acids via CD169 for cross-priming CD8$^+$ T cells (Backer et al., 2010; van Dinther et al., 2018). In agreement with these earlier studies, we found that activation of DCs, monitored by surface expression of CD80, was reduced in the CD11c^{hi} DCs and CD11c$^+$CD8α$^+$ cDC1 population in the absence of CD169 (Figures 7A and 7B). These data suggested that retrovirus-binding CD169$^+$ macrophages likely interacted with DCs and activated them. To test this possibility, we immunostained splenic sections 2 hr after r.o. administration of Gag-GFP-labeled retroviral particles. The images revealed that CD11c$^+$ and XCR1$^+$ DCs were in close proximity to retrovirus capturing CD169$^+$ MMM (Figure S7; Video S2). XCR1$^+$ cDC1s are known for their CD8$^+$ T cell cross-priming abilities and require the transcription factor *Batf3* for their development (Hildner et al., 2008). We therefore tested the contribution of cDC1 during FVC infection (r.o.) using *Batf3*$^{-/-}$ mice. FVC-infected cell numbers were significantly elevated in spleens of *Batf3*$^{-/-}$ mice compared with B6 and phenocopied CD8$^+$ T cell-depleted B6 mice (Figure 7C). These data revealed that cross-priming *Batf3*-dependent cDC1s contributed to FVC-specific CTL activity and control of FVC infection. The higher levels of FVC infection in CD169$^{-/-}$ compared with *Batf3*$^{-/-}$ mice are consistent with our hypothesis that CD169 orchestrates both efficient capture of blood-borne retroviruses to limit virus dissemination within the spleen and induction of effective CD8$^+$ CTL response by collaborating with cDC1s.

DISCUSSION

Previously we were able to demonstrate retrovirus spread through cell-cell contacts of transsynaptic and virological synapses within pLNs (Sewald et al., 2015). Here, using FTY720 to

Figure 6. CD8$^+$ CTL Response Is Compromised in CD169$^{-/-}$ Mice

(A) Comparison of percent CD8$^+$ T cell population in the spleen of B6 and CD169$^{-/-}$ mice (n = 4) after i.p. administration of CD8α T cell depleting antibodies.

(B) FVC-infected cells in the spleen of B6 and CD169$^{-/-}$ mice (n = 4, 8 dpi, 2,500 SFFU s.c.) with and without CD8 T cell depletion for an experiment as in (A).

(C) Experimental design for estimating *in vivo* CTL activity using a 1:1 ratio of FrMLV Gag peptide pulsed dsRed$^+$ and non-pulsed GFP$^+$ splenocytes in FVC-infected (r.o., 2,500 SFFU) mice.

(D) Representative FACS plots showing comparative killing of Gag peptide pulsed dsRed$^+$ splenocytes in uninfected (n = 5) and infected B6.*Fv2*$^{s/s}$ (n = 10) and B6.*Fv2*$^{s/s}$CD169$^{-/-}$ (n = 5) mice for an experiment as in (C).

(E) The graph in the left panel shows the ratio of non-pulsed to pulsed peptide cells in uninfected and infected mice for the experiment shown in (D) in pLN and spleen. The right panel shows specific CTL killing activity of peptide-pulsed cells after normalization to uninfected mice.

(F) Specific CTL activity determined using *in vitro* assay at indicated effector-to-target ratios using purified CD8$^+$ T cells from spleens of uninfected or infected B6 or CD169$^{-/-}$ mice (7 dpi, 2,500 SFFU r.o.). 1:1 ratio of peptide pulsed dsRed$^+$ and non-pulsed GFP$^+$ splenocytes were used as targets and CTL activity monitored as in (D) after culturing cells for 48 hr.

(G–K) 2 × 10^6 splenocytes from FVC-infected B6.*Fv2*$^{s/s}$ (n = 5) and B6.*Fv2*$^{s/s}$CD169$^{-/-}$ (n = 5) (8 dpi, 2,500 SFFU s.c.) or uninfected mice were cultured *in vitro* with 6 μM Gag peptide for 15–18 hr. The plots show a comparison of cells that stained positive for indicated markers in the CD8$^+$ T cell population.

(L) Experimental design to test the *in vivo* efficacy of adoptively transferred primed CD8$^+$ T cells from B6 or CD169$^{-/-}$ mice to target FVC-infected cells.

(M) FVC-infected cells in the spleen of CD169$^{-/-}$ mice for an experiment depicted in (L) (n = 4, 7 dpi, 2,500 SFFU r.o.). CD169$^{-/-}$ mice that did not receive exogenous CD8$^+$ T cells were used as control.

p values derived from non-parametric Mann-Whitney test; mean values denoted by horizontal line, error bars denote SD. See also Figures S4–S6.

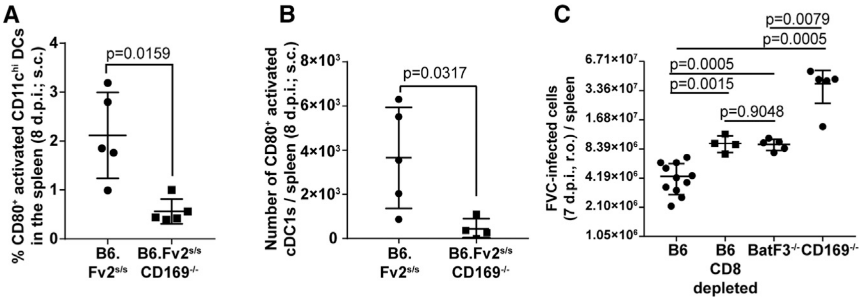

Figure 7. CD169 Plays a Crucial Role in Activating cDC1s to Elicit Effective $CD8^+$ T Cell Response during FVC Infection

(A and B) $CD80^+$-activated dendritic cells in $CD11c^{hi}$ (A) and $CD11c^+CD8\alpha^+$ (cDC1) (B) populations from splenocytes of FVC-infected B6.*Fv2*$^{s/s}$ (n = 5) and B6.*Fv2*$^{s/s}$CD169$^{-/-}$ (n = 5) (8 dpi, 2,500 SFFU s.c.).
(C) FVC-infected cells in the spleens of B6 (with and without CD8 T cell depletion), *Batf3*$^{-/-}$, and CD169$^{-/-}$ mice (n = 4, 7 dpi, 2,500 SFFU r.o.).
p values derived from non-parametric Mann-Whitney test; mean values denoted by horizontal line; error bars denote SD. Scale bars as indicated.
See also Figure S7 and Video S2.

block lymphocyte emigration, we show that retroviruses initially exploit lymph and blood flow (Figures 1 and S1A–S1D) to spread in cell-free mode. Host factors such as CD169 expressed on sentinel macrophages in the SCS and MZ located at the fluid-tissue interface played a crucial role in the transition of virus dissemination from a cell-free to cell-associated mode by capturing them and promoting *trans*-infection of permissive lymphocytes. Once the infection was established, retroviruses also exploited the migratory behavior of infected lymphocytes for their dissemination (Figures S1E–S1G). These data indicated that retroviruses are adept at using the advantages of both modes of transmission to facilitate their spread within the host.

How CD169-mediated capture and dissemination-limiting events affect retroviral pathogenesis was unknown. Here we utilized the non-pathogenic and pathogenic nature of two retroviruses, FrMLV and FVC, respectively, to study this aspect. For both viruses, CD169 reduced systemic viral dissemination by efficiently capturing free retroviruses from lymph and promoted infection at the pLN after s.c. challenge. Despite virus escape from pLN and higher viral loads in the spleen, FrMLV infection was lower in the spleens of CD169$^{-/-}$ compared with B6 mice. These data implied that regardless of the tissue, FrMLV relied on CD169-mediated capture event to efficiently infect target lymphocytes and corroborated the infection-promoting role for CD169 from our earlier study (Sewald et al., 2015). Our data are consistent with non-pathogenic FrMLV having evolved over a million years to exploit CD169-mediated capture for promoting infection of its native host (Figure 1G). Incorporation of sialic acid ligands to exploit CD169-mediated sequestration appears to be an evolutionary choice, as all enveloped viruses do not co-opt this mechanism. HIV-2 does not efficiently incorporate sialic acids and hence cannot exploit CD169 to promote its infection (Kijewski et al., 2016).

The observed infection-promoting function for $CD169^+$ SCS macrophages was contrary to that seen for other viruses such as murid herpesvirus-4 (MuHV-4) and VSV (Frederico et al., 2015; Iannacone et al., 2010). SCS macrophages were readily infected by lymph-borne MuHV-4 and protected target B cells from infection as it usurped the incoming virus into a non-amplifying pathway. VSV similarly infected $CD169^+$ SCS macrophages to initiate a type I IFN response that prevented lethal virus spread to the central nervous system. Thus, the frontline position of SCS macrophages posed a significant barrier for both MuHV-4 and VSV to infect their target cells contrary to FrMLV (Sewald et al., 2015). Importantly, analogous to its infection-hindering role for MuHV-4 and VSV, CD169 assumed a protective role during the pathogenic FVC infection. In the absence of CD169, FVC displayed enhanced infection at the spleen and led to accelerated death in susceptible BALB/cJ mice. This surprising opposite outcome was in part due to the expanded tropism of FVC that can also productively infect and proliferate in erythroblasts. Lack of CD169-mediated virus-filtering activity in the MZ provided FVC enhanced access to the red pulp, which is rich in target erythroblast population, resulting in elevated levels of infection. In contrast, FrMLV cannot productively infect erythroblasts and required CD169 expression to promote its infection of permissive lymphocytes. Our data highlight an interesting facet of host-pathogen interaction whereby retroviruses co-evolved to hijack the surveillance function of $CD169^+$ macrophages for their efficient infection and spread in mice. Despite this exploitation, the protective function of the $CD169^+$ macrophages dominate when a pathogenic virus arises with an expanded tropism. Thus, by comparing FrMLV and FVC we were able to reveal both infection-promoting and protective roles for CD169 on sentinel macrophages during retrovirus infection.

A protective role for CD169, though similar to other pathogens as noted in previous studies, could not be attributed solely to the lectin CD169 (Farrell et al., 2015, 2016; Gupta et al., 2016; Honke et al., 2012; Iannacone et al., 2010; Kastenmuller et al., 2012; Sagoo et al., 2016). Many of the previous studies employed clodronate liposomes or diphtheria toxin receptor-based strategies that eliminated SCS or MMM macrophage populations entirely. Thus, the strength of our work over previous studies is that we document a direct role of CD169 per se with an intact macrophage layer.

In addition to regulating dissemination, CD169 expression could also influence several immune related functions that are orchestrated by sentinel macrophages. CD169-expressing SCS macrophages were shown to capture tumor-derived vesicles and exosomes from the lymph, preventing them from accessing the B cell follicular area. This step protected the host from deleterious effects of tumor-promoting humoral immunity (Pucci et al., 2016). However, we did not observe significant differences in FVC-specific humoral immune $CD4^+$ T cell responses when CD169 was absent (Figures S4 and S5). $CD169^+$ macrophages can also promote activation of tumor-specific $CD8^+$ T cells by promoting cross-presentation of tumor-derived antigens (Asano et al., 2011). We found that CD169 expression on MZ macrophages was required to elicit

an effective FVC-specific CD8+ T cytotoxic response (Figures 6 and 7) for elimination of infected cells. When CD169 was absent, we observed an overall reduction in numbers of IFN-γ-expressing CD8+ T cells stimulated by PMA/ionomycin or Gag-specific peptide (Figures S6 and 6J). A weak CD8+ T cell response in CD169$^{-/-}$ mice was associated with compromised activation of cDCs. Given that CD169+ MMM capture incoming retroviruses, efficient activation could be limited to interrogating DCs. Unlike VSV, retrovirus-laden CD169+ macrophages are not infected early during infection (Honke et al., 2012; Sewald et al., 2015), and hence suggested a role for naturally proficient cross-presenting cDC1s in mounting a rapid CD8+ CTL response. Indeed, we observed that FVC-specific CD8+ T cell responses were severely compromised in *Batf3*$^{-/-}$ mice lacking cDC1 cells and phenocopied mice in which CD8+ T cells were depleted (Figure 7C). These data suggested a crucial contribution of CD169 in cross-presentation of captured natural ligands such as retroviruses via cDC1s to elicit effective CD8+ T cell response. Our data imply that exploration of CD169-blockade-based strategy to reduce HIV-1 acquisition needs to be considered with caution as it can also compromise antigen cross-presentation and/or alter protective CD8+ T cell responses. Our data are in agreement with a recent study in which CD169 expressed on MZ macrophages was shown to bind sialic acids on the cell surface of interrogating cDC1s for cross-presentation of antigens to promote effective CD8+ T cell responses (van Dinther et al., 2018). Our studies highlight the emerging importance of the I-type lectin CD169 expressed on sentinel macrophages in curbing systemic dissemination of retroviruses and promoting cell-cell interactions for orchestrating effective immune responses. A detailed understanding of how the early events shape the outcome of viral infections is therefore required to inform the design of effective antiviral and vaccination strategies.

STAR★METHODS

Detailed methods are provided in the online version of this paper and include the following:

- KEY RESOURCES TABLE
- CONTACT FOR REAGENT AND RESOURCE SHARING
- EXPERIMENTAL MODEL AND SUBJECT DETAILS
  - Mice
  - Virus Production and Titration
- METHOD DETAILS
  - Retrovirus Infection and Treatment Conditions
  - Single Cell Preparation from Mouse Tissue
  - Monitoring Virus Particle Flow
  - Glycogag-Alexa Conjugates
  - Flow Cytometric Analyses
  - DFJ8 Co-culture for Determining Infectivity
  - Cryo-Immunohistology of Spleen
  - Sample Preparation for Electron Microscopy
  - Electron Microscopy and Dual-Axis Tomography
  - Neutralizing Antibody Titer
  - FVC-Specific CD4+ T Cell Proliferation
  - Type I Interferon Response
  - Measuring CTL Activity *In Vivo* and *In Vitro*
  - CD8+ T Cell Analyses
  - CD8+ T Cell Adoptive Transfer
- QUANTIFICATION AND STATISTICAL ANALYSIS

SUPPLEMENTAL INFORMATION

Supplemental Information includes seven figures and two videos and can be found with this article online at https://doi.org/10.1016/j.chom.2018.11.011.

ACKNOWLEDGMENTS

This work was supported by NIH grants R01 CA098727 to W.M. and P50GM082545 to W.M. and P.B.J.; the Flow Cytometry Shared Resource of the Yale Cancer Center P30 CA016359, Yale Center for Cellular and Molecular Imaging S10 OD020142, R21 AI112418 to M.L.S.; funding from the Francis Crick Institute (FC001099) to G.K.; and a fellowship from the China Scholarship Council – Yale World Scholars to R.P. We thank Stephanie Eisenbarth for providing *Batf3*$^{-/-}$ mice, and Frank Malik, Leonard Evans, and Kim Hasenkrug for suggestions and providing reagents.

AUTHOR CONTRIBUTIONS

P.D.U. and R.P. contributed equally to executing experiments, data analysis, and figure generation. P.D.U., R.P., and W.M. shared the conceptualization, experimental design, interpretation, and manuscript preparation. K.A.H. generated constructs and mouse strains. M.S.L. carried out electron tomography. J.D.V. helped with cryo-sectioning of tissues. B.S.B., M.L.S., and G.K. provided all the reagents, protocols, and EF4.1 mouse strains for carrying out FVC work. M.L.S., X.S., P.J.B., and G.K. contributed to interpretation and discussion of the work.

DECLARATION OF INTERESTS

The authors declare no competing interests.

Received: June 10, 2018
Revised: October 8, 2018
Accepted: November 5, 2018
Published: December 27, 2018

REFERENCES

Alberti, M.O., Jones, J.J., Miglietta, R., Ding, H., Bakshi, R.K., Edmonds, T.G., Kappes, J.C., and Ochsenbauer, C. (2015). Optimized replicating renilla luciferase reporter HIV-1 utilizing novel internal ribosome entry site elements for native nef expression and function. AIDS Res. Human Retroviruses *31*, 1278–1296.

Antunes, I., Tolaini, M., Kissenpfennig, A., Iwashiro, M., Kuribayashi, K., Malissen, B., Hasenkrug, K., and Kassiotis, G. (2008). Retrovirus-specificity of regulatory T cells is neither present nor required in preventing retrovirus-induced bone marrow immune pathology. Immunity *29*, 782–794.

Arnon, T.I., Horton, R.M., Grigorova, I.L., and Cyster, J.G. (2013). Visualization of splenic marginal zone B-cell shuttling and follicular B-cell egress. Nature *493*, 684–688.

Asano, K., Nabeyama, A., Miyake, Y., Qiu, C.H., Kurita, A., Tomura, M., Kanagawa, O., Fujii, S., and Tanaka, M. (2011). CD169-positive macrophages dominate antitumor immunity by crosspresenting dead cell-associated antigens. Immunity *34*, 85–95.

Backer, R., Schwandt, T., Greuter, M., Oosting, M., Jungerkes, F., Tuting, T., Boon, L., O'Toole, T., Kraal, G., Limmer, A., et al. (2010). Effective collaboration between marginal metallophilic macrophages and CD8+ dendritic cells in the generation of cytotoxic T cells. Proc. Natl. Acad. Sci. U S A *107*, 216–221.

Bernhard, C.A., Ried, C., Kochanek, S., and Brocker, T. (2015). CD169+ macrophages are sufficient for priming of CTLs with specificities left out by cross-priming dendritic cells. Proc. Natl. Acad. Sci. U S A *112*, 5461–5466.

Cerutti, A., Cols, M., and Puga, I. (2013). Marginal zone B cells: virtues of innate-like antibody-producing lymphocytes. Nat. Rev. Immunol. *13*, 118–132.

Chesebro, B., Miyazawa, M., and Britt, W.J. (1990). Host genetic control of spontaneous and induced immunity to Friend murine retrovirus infection. Annu. Rev. Immunol. *8*, 477–499.

Chesebro, B., Wehrly, K., Cloyd, M., Britt, W., Portis, J., Collins, J., and Nishio, J. (1981). Characterization of mouse monoclonal antibodies specific for Friend murine leukemia virus-induced erythroleukemia cells: friend-specific and FMR-specific antigens. Virology *112*, 131–144.

Chu, J., Oh, Y., Sens, A., Ataie, N., Dana, H., Macklin, J.J., Laviv, T., Welf, E.S., Dean, K.M., Zhang, F., et al. (2016). A bright cyan-excitable orange fluorescent protein facilitates dual-emission microscopy and enhances bioluminescence imaging in vivo. Nat. Biotechnol. *34*, 760–767.

Constantinescu, S.N., Wu, H., Liu, X., Beyer, W., Fallon, A., and Lodish, H.F. (1998). The anemic Friend virus gp55 envelope protein induces erythroid differentiation in fetal liver colony-forming units-erythroid. Blood *91*, 1163–1172.

Farrell, H.E., Bruce, K., Lawler, C., Cardin, R.D., Davis-Poynter, N.J., and Stevenson, P.G. (2016). Type 1 interferons and NK cells limit murine cytomegalovirus escape from the lymph node subcapsular sinus. PLoS Pathog. *12*, e1006069.

Farrell, H.E., Davis-Poynter, N., Bruce, K., Lawler, C., Dolken, L., Mach, M., and Stevenson, P.G. (2015). Lymph node macrophages restrict murine cytomegalovirus dissemination. J. Virol. *89*, 7147–7158.

Frederico, B., Chao, B., Lawler, C., May, J.S., and Stevenson, P.G. (2015). Subcapsular sinus macrophages limit acute gammaherpesvirus dissemination. J. Gen. Virol. *96*, 2314–2327.

Gupta, P., Lai, S.M., Sheng, J., Tetlak, P., Balachander, A., Claser, C., Renia, L., Karjalainen, K., and Ruedl, C. (2016). Tissue-resident CD169(+) macrophages form a crucial front line against plasmodium infection. Cell Rep. *16*, 1749–1761.

Hasenkrug, K.J., and Chesebro, B. (1997). Immunity to retroviral infection: the friend virus model. Proc. Natl. Acad. Sci. U S A *94*, 7811–7816.

Hasenkrug, K.J., and Dittmer, U. (2000). The role of CD4 and CD8 T cells in recovery and protection from retroviral infection: lessons from the friend virus model. Virology *272*, 244–249.

Hildner, K., Edelson, B.T., Purtha, W.E., Diamond, M., Matsushita, H., Kohyama, M., Calderon, B., Schraml, B.U., Unanue, E.R., Diamond, M.S., et al. (2008). Batf3 deficiency reveals a critical role for CD8alpha^{+} dendritic cells in cytotoxic T cell immunity. Science *322*, 1097–1100.

Honke, N., Shaabani, N., Cadeddu, G., Sorg, U.R., Zhang, D.E., Trilling, M., Klingel, K., Sauter, M., Kandolf, R., Gailus, N., et al. (2012). Enforced viral replication activates adaptive immunity and is essential for the control of a cytopathic virus. Nat. Immunol. *13*, 51–57.

Iannacone, M., Moseman, E.A., Tonti, E., Bosurgi, L., Junt, T., Henrickson, S.E., Whelan, S.P., Guidotti, L.G., and von Andrian, U.H. (2010). Subcapsular sinus macrophages prevent CNS invasion on peripheral infection with a neurotropic virus. Nature *465*, 1079–1083.

Jin, J., Sherer, N.M., Heidecker, G., Derse, D., and Mothes, W. (2009). Assembly of the murine leukemia virus is directed towards sites of cell-cell contact. PLoS Biol. *7*, e1000163.

Junt, T., Moseman, E.A., Iannacone, M., Massberg, S., Lang, P.A., Boes, M., Fink, K., Henrickson, S.E., Shayakhmetov, D.M., Di Paolo, N.C., et al. (2007). Subcapsular sinus macrophages in lymph nodes clear lymph-borne viruses and present them to antiviral B cells. Nature *450*, 110–114.

Kastenmuller, W., Torabi-Parizi, P., Subramanian, N., Lammermann, T., and Germain, R.N. (2012). A spatially-organized multicellular innate immune response in lymph nodes limits systemic pathogen spread. Cell *150*, 1235–1248.

Kijewski, S.D.G., Akiyama, H., Feizpour, A., Miller, C.M., Ramirez, N.P., Reinhard, B.M., and Gummuluru, S. (2016). Access of HIV-2 to CD169-dependent dendritic cell-mediated trans infection pathway is attenuated. Virology *497*, 328–336.

Li, J.P., D'Andrea, A.D., Lodish, H.F., and Baltimore, D. (1990). Activation of cell growth by binding of Friend spleen focus-forming virus gp55 glycoprotein to the erythropoietin receptor. Nature *343*, 762–764.

Lilly, F. (1970). Fv-2: identification and location of a second gene governing the spleen focus response to Friend leukemia virus in mice. J. Natl. Cancer Inst. *45*, 163–169.

Logg, C.R., Logg, A., Tai, C.K., Cannon, P.M., and Kasahara, N. (2001). Genomic stability of murine leukemia viruses containing insertions at the Env-3' untranslated region boundary. J Virol. *75*, 6989–6998.

Marques, R., Antunes, I., Eksmond, U., Stoye, J., Hasenkrug, K., and Kassiotis, G. (2008). B lymphocyte activation by coinfection prevents immune control of friend virus infection. J. Immunol. *181*, 3432–3440.

Martin, F., and Kearney, J.F. (2002). Marginal-zone B cells. Nat. Rev. Immunol. *2*, 323–335.

Martinez-Pomares, L., and Gordon, S. (2012). CD169+ macrophages at the crossroads of antigen presentation. Trends Immunol. *33*, 66–70.

Matloubian, M., Lo, C.G., Cinamon, G., Lesneski, M.J., Xu, Y., Brinkmann, V., Allende, M.L., Proia, R.L., and Cyster, J.G. (2004). Lymphocyte egress from thymus and peripheral lymphoid organs is dependent on S1P receptor 1. Nature *427*, 355–360.

Meyer-Bahlburg, A., Andrews, S.F., Yu, K.O., Porcelli, S.A., and Rawlings, D.J. (2008). Characterization of a late transitional B cell population highly sensitive to BAFF-mediated homeostatic proliferation. J. Exp. Med. *205*, 155–168.

Miyazawa, M., Tsuji-Kawahara, S., and Kanari, Y. (2008). Host genetic factors that control immune responses to retrovirus infections. Vaccine *26*, 2981–2996.

Nowinski, R.C. (1976). Genetic control of natural immunity to ecotropic mouse leukemia viruses: immune response genes. Infect. Immun. *13*, 1098–1102.

Nguyen, A.W., and Daugherty, P.S. (2005). Evolutionary optimization of fluorescent proteins for intracellular FRET. Nat. Biotechnol. *23*, 355–360.

Oetke, C., Vinson, M.C., Jones, C., and Crocker, P.R. (2006). Sialoadhesin-deficient mice exhibit subtle changes in B- and T-cell populations and reduced immunoglobulin M levels. Mol. Cell. Biol. *26*, 1549–1557.

Oliff, A.I., Hager, G.L., Chang, E.H., Scolnick, E.M., Chan, H.W., and Lowy, D.R. (1980). Transfection of molecularly cloned Friend murine leukemia virus DNA yields a highly leukemogenic helper-independent type C virus. J. Virol. *33*, 475–486.

Oliver, A.M., Martin, F., Gartland, G.L., Carter, R.H., and Kearney, J.F. (1997). Marginal zone B cells exhibit unique activation, proliferative and immunoglobulin secretory responses. Eur. J. Immunol. *27*, 2366–2374.

Persons, D.A., Paulson, R.F., Loyd, M.R., Herley, M.T., Bodner, S.M., Bernstein, A., Correll, P.H., and Ney, P.A. (1999). Fv2 encodes a truncated form of the Stk receptor tyrosine kinase. Nat. Genet. *23*, 159–165.

Pucci, F., Garris, C., Lai, C.P., Newton, A., Pfirschke, C., Engblom, C., Alvarez, D., Sprachman, M., Evavold, C., Magnuson, A., et al. (2016). SCS macrophages suppress melanoma by restricting tumor-derived vesicle-B cell interactions. Science *352*, 242–246.

Quah, B.J., Wijesundara, D.K., Ranasinghe, C., and Parish, C.R. (2012). Fluorescent target array killing assay: a multiplex cytotoxic T-cell assay to measure detailed T-cell antigen specificity and avidity in vivo. Cytometry A *81*, 679–690.

Refaeli, Y., Van Parijs, L., Alexander, S.I., and Abbas, A.K. (2002). Interferon gamma is required for activation-induced death of T lymphocytes. J. Exp. Med. *196*, 999–1005.

Rosenberg, N., and Jolicoeur, P. (1997). Retroviral pathogenesis. In Retroviruses, J.M. Coffin, S.H. Hughes, and H.E. Varmus, eds. (Cold Spring Harbor Laboratory Press), pp. 475–586.

Sagoo, P., Garcia, Z., Breart, B., Lemaitre, F., Michonneau, D., Albert, M.L., Levy, Y., and Bousso, P. (2016). In vivo imaging of inflammasome activation reveals a subcapsular macrophage burst response that mobilizes innate and adaptive immunity. Nat. Med. *22*, 64–71.

Santiago, M.L., Montano, M., Benitez, R., Messer, R.J., Yonemoto, W., Chesebro, B., Hasenkrug, K.J., and Greene, W.C. (2008). Apobec3 encodes Rfv3, a gene influencing neutralizing antibody control of retrovirus infection. Science *321*, 1343–1346.

Saunderson, S.C., Dunn, A.C., Crocker, P.R., and McLellan, A.D. (2014). CD169 mediates the capture of exosomes in spleen and lymph node. Blood *123*, 208–216.

Schmittgen, T.D., and Livak, K.J. (2008). Analyzing real-time PCR data by the comparative C(T) method. Nat. Protoc. *3*, 1101–1108.

Sewald, X., Ladinsky, M.S., Uchil, P.D., Beloor, J., Pi, R., Herrmann, C., Motamedi, N., Murooka, T.T., Brehm, M.A., Greiner, D.L., et al. (2015). Retroviruses use CD169-mediated trans-infection of permissive lymphocytes to establish infection. Science *350*, 563–567.

Shao, L., Takeda, K., Kato, S., Mori, S., and Kodama, T. (2015). Communication between lymphatic and venous systems in mice. J. Immunol. Methods *424*, 100–105.

van Dinther, D., Veninga, H., Iborra, S., Borg, E.G.F., Hoogterp, L., Olesek, K., Beijer, M.R., Schetters, S.T.T., Kalay, H., Garcia-Vallejo, J.J., et al. (2018). Functional CD169 on macrophages mediates interaction with dendritic cells for CD8(+) T cell cross-priming. Cell Rep. *22*, 1484–1495.

Yap, M.W., Colbeck, E., Ellis, S.A., and Stoye, J.P. (2014). Evolution of the retroviral restriction gene Fv1: inhibition of non-MLV retroviruses. PLoS Pathog. *10*, e1003968.

Yoon, S.M., Namkung, W., and Lee, J. (2013). A comparison of Ypet and firefly luciferase as reporter proteins for high-throughput screening. Biosci. Biotechnol. Biochem. *77*, 2328–2330.

Zouali, M., and Richard, Y. (2011). Marginal zone B-cells, a gatekeeper of innate immunity. Front Immunol. *2*, 63.

STAR★METHODS

KEY RESOURCES TABLE

REAGENT or RESOURCE	SOURCE	IDENTIFIER
Antibodies		
Ultra-LEAF purified anti-mouse CD169 (3D6.112)	BioLegend	Cat # 94019
Ultra-LEAF purified Rat IgG2a isotype control antibody (RTK2758)	BioLegend	Cat # 400543, RRID: AB_11148951
Fc block anti mouse-CD16/CD32 (93)	BioLegend	Cat # 101302, RRID: AB_312801
Anti-MLV Glycogag (mab34)	Santiago Lab/ Bruce Chesebro	Recognizes MLV (MA, p15) part of Glycogag (Chesebro et al., 1981)
Anti-MLV Gag p30 hybridoma (R187)	ATCC	Cat # CRL-1912
AF647 anti-MLV Glycogag (mab34)	Prepared in this work	N/A
FITC anti-mouse CD19 (6D5)	BioLegend	Cat # 115505, RRID: AB_313640
PE/Cy7 anti-mouse CD19(6D5)	BioLegend	Cat # 115519, RRID: AB_313654
APC anti-mouse CD4(RM4-5)	BioLegend	Cat # 100515, RRID: AB_312718
AF647 anti-mouse CD4 (GK1.5)	BioLegend	Cat # 100426, RRID: AB_493519
PE/Cy7 anti-mouse CD4 (GK1.5)	BioLegend	Cat # 100421, RRID: AB_312706
APC/Cy7 anti-mouse CD3ε (145-2C11)	BioLegend	Cat # 100329, RRID: AB_1877171
PE anti-mouse CD71 (RI7217)	BioLegend	Cat # 113807, RRID: AB_313568
APC/Cy7 anti-mouse TER-119 (TER-119)	BioLegend	Cat # 116223, RRID: AB_2137788
AF647 anti-mouse CD169 (3D6.112)	BioLegend	Cat # 142407, RRID: AB_2563620
PE anti-mouse CD169 (3D6.112)	BioLegend	Cat # 142403, RRID: AB_10915470
AF594 anti-mouse CD169 (3D6.112)	BioLegend	Cat # 142416, RRID: AB_2565620
FITC anti-mouse CD21/CD35 (CR2/CR1) (7E9)	BioLegend	Cat # 123407, RRID: AB_940403
PE anti-mouse CD23 (B3B4)	BioLegend	Cat # 101607, RRID: AB_312832
eFluor450 anti-mouse IgD, eBioscience (11-26c(11-26))	Invitrogen	REF # 48-5993-80, RRID: AB_1272239
Dylight 550 goat anti-mouse IgM cross-abosorbed secondary antibody	Invitrogen	Cat # SA5-10151, RRID: AB_2556731
PE anti-mouse CD45.1 (A20)	BioLegend	Cat # 110707, RRID: AB_313496
FITC anti-mouse CD45.2 (104)	BioLegend	Cat # 109805, RRID: AB_313442
APC anti-mouse/human CD44 (IM7)	BioLegend	Cat # 103011, RRID: AB_312962
InVivoMAb anti-mouse CD8α (YTS 169.4)	Bio X cell	Cat # BE0117, RRID: AB_10950145
AF488 anti-mouse CD8α (53-6.7)	BioLegend	Cat # 100723, RRID: AB_389304
AF647 anti-mouse CD107A (LAMP-1) (1D4B)	BioLegend	Cat #121609, RRID: AB_571990
PE anti-mouse Granzyme A (3G8.5)	BioLegend	Cat # 149703, RRID: AB_2565309
PE Anti-human/mouse Granzyme B Recombinant (QA16A02)	BioLegend	Cat # 372207, RRID: AB_2687031
PE anti-mouse IFNγ (XMG1.2)	BioLegend	Cat # 505807, RRID: AB_315401
PE anti-mouse CD279 (PD-1) (RMP1-30)	BioLegend	Cat # 109103, RRID: AB_313420
PE anti-mouse CD80 (16-10A1)	BioLegend	Cat # 104707, RRID: AB_313128
APC anti-mouse CD80 (16-10A1)	BioLegend	Cat # 104713, RRID: AB_313134
AF647 anti-mouse CD11c (N418)	BioLegend	Cat # 117314, RRID: AB_492850
APC/Cy7 anti-mouse CD11c (N418)	BioLegend	Cat # 117323, RRID: AB_830646
Alexa Fluor 647 anti-mouse/rat XCR1 (ZET)	BioLegend	Cat # 148213, RRID: AB_2564368
Alexa Fluor 647 anti-mouse CD11c	BioLegend	Cat # 117312, RRID: AB_389328

(*Continued on next page*)

Continued		
REAGENT or RESOURCE	SOURCE	IDENTIFIER
Bacterial and Virus Strains		
Lactate dehydrogenase-elevating virus (LDV)-free FVC	Generated in this work by passaging the virus in BALB/cJ mice	N/A
FrMLV copackaged with MLV LTR Antares	Generated in this work	N/A
FrMLV co-packaged with MLV LTR GFP	Mothes Lab, Yale University	N/A
FVCYpet	Generated in this work	N/A
FVC GFP	Generated in this work	N/A
Chemicals, Peptides, and Recombinant Proteins		
Liberase TL Research Grade	Sigma-Aldrich	Cat# 5401020001
DNAse I recombinant, RNAse-free	Roche	Ref # 04716728001
RPMI medium 1640 (1X)	Life technologies	Ref # 11875-093
Fetal bovine serum	Atlanta Biologicals	Cat # S11550
MEM Non-essential amino acid (NEAA) solution (100X)	Life technologies	Ref # 11140-050
Penicillin-streptomycin solution (10,000 U/ml)	Life technologies	Ref # 15140122
Sodium pyruvate (100 mM)	Life technologies	Ref # 11360-070
2-Mercaptoethanol	Sigma-Aldrich	Cat # M3148
L-Glutamine (200mM)	Life technologies	Ref # 25030-081
Red blood cell lysis buffer-Hybri-Max	Sigma-Aldrich	Cat # R7757-100ML
RBC Lysis Buffer (10X)	BioLegend	Cat # 420301
Dulbecco's Phosphate Buffered Saline (DPBS) 1X	Life technologies	Ref # 14190-144
Hybridoma-SFM	Gibco	Cat # 12045-076
Ultra-low IgG FBS	Life technologies	Cat # 16250-086
Bovine Serum Albumin (BSA)	Sigma-Aldrich	Cat# A9647-100G CAS: 9048-46-8
Accutase	Biolegend	Cat # 423201
0.05% Trypsin-EDTA (1X)	Life Technologies	Cat # 25300-054
K3 EDTA 15% Solution	Fisher Scientific	Cat # BD 366450
Gelatin (Teleostean gelatin) Type A	Sigma-Aldrich	Cat # G7041 CAS: 9000-70-8
Triton-X 100 t-octyl phenoxy polyethoxyethanol	American Bioanalytical	Cat # AB02025-00500 CAS: 9002-93-1
PMA (phorbol 12-myristate-13-acetate)	Sigma	Cat # 19-144
Ionomycin	Sigma	Cat # I3909-1ML
GolgiStop	BD Biosciences	Cat # 554724
Brefeldin A	Sigma-Aldrich	Cat # B7651-5MG CAS: 20350-15-6
Paraformaldehyde (PFA)	Electron Microscopy Sciences	Cat # 19200 CAS: 30525-89-4
Rat serum	Stemcell Biotechnologies	Cat # 13551
L-lysine Monohydrochloride	Sigma-Aldrich	Cat # L1262
Sodium (meta)periodate	Sigma-Aldrich	Cat # 30323-100G CAS: 7790-28-5
Sucrose/ α-D-glucopyranosyl-β-D-fructofuranoside	americanBIO	Ref # AB01900-01000 CAS: 57-50-1
Tissue-Tek O.C.T Compound	Sakura	Cat # 4583
Fc receptor blocker	Innovex	Cat # NB335
ProLong Gold antifade reagent	Invitrogen	Cat # P36934
Glutaraldehyde	Electron Microscopy Sciences	Cat # 16220 CAS: 111-30-8
Sodium cacodylate trihydrate	Electron Microscopy Sciences	Cat #12300
Ficoll	Sigma-Aldrich	Cat #F2878-100g
Osmium tetroxide	Electron Microscopy Sciences	Cat #19110

(*Continued on next page*)

Continued

REAGENT or RESOURCE	SOURCE	IDENTIFIER
Uranyl acetate	Electron Microscopy Sciences	Cat #22400
Acetone, EM-Grade, Glass-Distilled	Electron Microscopy Sciences	Cat #10015
Epon-Araldite resin	Electron Microscopy Sciences	Cat #13940
Lead citrate	Electron Microscopy Sciences	Cat #17800 CAS: 512-26-5
Gold beads (10 nm)	Ted Pella	Cat. #15703-1
Bouin's solution	Sigma-Aldrich	Cat # HT10132-1L
FTY720	Cayman Chemical	Cat # 10006292 CAS: 162359-56-0
Dimethyl sulfoxide (DMSO)	Sigma-Aldrich	Cat # D2650-5X5ML CAS: 67-68-5
Sodium azide	Sigma-Aldrich	Cat # S-8032 EC No: 247-852-1
Sodium phosphate, Monobasic, Monohydrate, Crystal ($NaH_2PO_4 \cdot H_2O$)	J.T.Baker	Cat # 3818-01 CAS: 10049-21-5
Sodium phosphate, Dibasic, Anhydrous (Na_2HPO_4)	J.T.Baker	Cat # 3828-01 CAS: 7558-79-4
Glycine	American Bioanalytical	Cat # AB00730-01000 CAS: 56-40-6
Passive lysis buffer (5X)	Promega	Cat # E194A
Guinea pig complement	MP Biomedical	Cat # 55854
DNase inactivation reagent	Ambion	Cat # 8173G
MLV specific peptide (GK1754) (KKCCLCLTVFL)	Genscript	N/A
FrMLV Gag peptide (CCLCLTVFL)	Peptide 2.0	N/A
Critical Commercial Assays		
Mix-n-Stain CF 488A Antibody Labeling Kit (50-100µg)	Sigma-Aldrich	Cat # MX488AS100 SIGMA
Mix-n-Stain CF 647 Antibody Labeling Kit (50-100µg)	Sigma-Aldrich	Cat # MX647S100 SIGMA
Nano-Glo Luciferase Assay System	Promega	Cat # N1120
KAPA SYBR FAST qPCR Master Mix (2X) Kit	KAPA Biosystems	Cat # KK4600 and KK4601
Ambion DNase I (RNase-free)	Thermo Fisher Scientific	Cat # AM2222
RNeasy Mini Kit (50)	Qiagen	Cat #/ID 74104
qScript cDNA Synthesis Kit	Quanta Biosciences	Cat # 95047-100
Negative selection mouse CD4$^+$ T cell enrichment kit	Stemcell technologies	Cat # 19752A
MojoSort mouse CD8 T cell isolation kit	BioLegend	Cat # 480008
Experimental Models: Cell Lines		
Rat hybridoma mAb34	Santiago Lab/ Bruce Chesebro	Recognizes MLV (MA, p15) part of Glycogag (Chesebro et al., 1981)
HEK293	ATCC	Cat # CRL-1573
S49.1	ATCC	Cat # TIB-28
DFJ8	Mothes Lab (From Jim Cunningham, Dana Farber)	N/A
Experimental Models: Organisms/Strains		
C57BL/6J (B6)	The Jackson Laboratory	The Jackson Laboratory Stock No: 000664
BALB/cJ	The Jackson Laboratory	The Jackson Laboratory Stock No: 000651
CD169$^{-/-}$ (B6 background)	Paul Crocker, University of Dundee UK	N/A
B6.A-*Fv2*$^{s/s}$ (B6 background)	The Francis Crick Institute, UK	Colony ID: GKAF
B6.A-*Fv2*$^{s/s}$CD169$^{-/-}$ (B6 background)	Generated in this work	N/A
Ifnar1$^{-/-}$ (B6 background)	Iwasaki Lab, Yale University	MMRRC Stock No: 32045-JAX
Ifnar1$^{-/-}$CD169$^{-/-}$ (B6 background)	Generated in this work	N/A

(*Continued on next page*)

Continued

REAGENT or RESOURCE	SOURCE	IDENTIFIER
NagyDsRed.T3 (B6 background)	The Jackson Laboratory	Jackson Laboratory Stock No: 006051
UBI-GFP (B6 background)	The Jackson Laboratory	Jackson Laboratory Stock No: 004353
F-MuLV env-specific TCR-transgenic mouse (EF4.1 strain TCRβ transgenic mouse)	The Francis Crick Institute, UK	Colony ID: GKAA
Batf3$^{-/-}$ (B6 background)	Eisenbarth Lab, Yale University	Jackson Laboratory Stock No: 013755
Oligonucleotides		
Mouse Actin, F: 5'-CATGTAGATGCACGACTAGCTTC-3' R: 5'-GTTTCCTTGTTTAGCAGAACAGC-3'	Yale School of Medicine, W. M. Keck Foundation, Oligo Synthesis Resource	N/A
Mouse *IFNB1*, F: 5'-CTGGCTTCCATCATGAACAA-3' R: 5'-AGAGGGCTGTGGTGGAGAA-3'	Yale School of Medicine, W. M. Keck Foundation, Oligo Synthesis Resource	N/A
Mouse *IFNA2*, F: 5'-TCTGTGCTTTCCTCGTGATG-3' R: 5'-TTGAGCCTTCTGGATCTGCT-3'	Yale School of Medicine, W. M. Keck Foundation, Oligo Synthesis Resource	N/A
Mouse *IFNA4*, F: 5'-GCAGAAGTCTGGAGAGCCCTC-3' R: 5'-TGAGATGCAGTGTTCTGGTCC-3'	Yale School of Medicine, W. M. Keck Foundation, Oligo Synthesis Resource	N/A
Mouse *IFITM3*, F: 5'-CTGAAGGGGAGCGATTGATT-3' R: 5'-AACGGCACATGACCAAAGAGTAGA-3'	Yale School of Medicine, W. M. Keck Foundation, Oligo Synthesis Resource	N/A
Mouse *IRF7*, F: 5'-GCCAGGAGCAAGACCGTGTT-3' R: 5'-TGCCCCACCACTGCCTGTA-3'	Yale School of Medicine, W. M. Keck Foundation, Oligo Synthesis Resource	N/A
Mouse *ISG15*, F: 5'-GATTGCCCAGAAGATTGGTG -3' R: 5'-TCTGCGTCAGAAAGACCTCA-3'	Yale School of Medicine, W. M. Keck Foundation, Oligo Synthesis Resource	N/A
Mouse *VIG1*, F: 5'-AACCCCCGTGAGTGTCAACTA-3' R: 5'-AACCAGCCTGTTTGAGCAGAA-3'	Yale School of Medicine, W. M. Keck Foundation, Oligo Synthesis Resource	N/A
Mouse *GBP4*, F: 5'-TGGGGGACACAGGCTCTACA-3' R: 5'-GCCTGCAGGATGGAACTCTCAA-3'	Yale School of Medicine, W. M. Keck Foundation, Oligo Synthesis Resource	N/A
Mouse *CXCL10*, F: 5'-CCAAGTGCTGCCGTCATTTTC-3' R: 5'-GGCTCGCAGGGATGATTTCAA-3'	Yale School of Medicine, W. M. Keck Foundation, Oligo Synthesis Resource	N/A
Mouse *STAT1*, F: 5'-CACATTCACATGGGTGGAAC-3' R: 5'-TCTGGTGCTTCCTTTGGTCT-3'	Yale School of Medicine, W. M. Keck Foundation, Oligo Synthesis Resource	N/A
Mouse *STAT2*, F: 5'-ACCAGTGGGACCACTACAGC-3' R: 5'-ATCTCAAGCTGCTGGCTCTC-3'	Yale School of Medicine, W. M. Keck Foundation, Oligo Synthesis Resource	N/A
Mouse *IL10*, F: 5'-CTCTTACTGACTGGCATGAGGAT-3' R: 5'-GAGTCGGTTAGCAGTATGTTGT-3'	Yale School of Medicine, W. M. Keck Foundation, Oligo Synthesis Resource	N/A
Mouse *25OAS*, F: 5'-ACTGTCTGAAGCAGATTGCG-3' R: 5'-TGGAACTGTTGGAAGCAGTC-3'	Yale School of Medicine, W. M. Keck Foundation, Oligo Synthesis Resource	N/A
Mouse *MX1*, F: 5'-AACCCTGCTACCTTTCAA-3' R: 5'-AAGCATCGTTTTCTCTATTTC-3'	Yale School of Medicine, W. M. Keck Foundation, Oligo Synthesis Resource	N/A
Recombinant DNA		
pLRB303-FrMLV	Mothes Lab, Yale University	N/A
pMMP-LTR-GFP	Mothes Lab, Yale University	N/A
pMIG-Antares	Generated in this work	N/A
pLRB303-FrMLVYpet	Generated in this work	N/A
pBR322-SFFV LS	Leonard Evans (NIH)	N/A
pLRB303-SFFV GFP	Generated in this work	N/A
pLRB303-GagGFP	Mothes Lab, Yale University	(Jin et al., 2009)
MLV GagPol	Mothes Lab, Yale University	N/A
pcDNA3-FrMLV Env	Mothes Lab, Yale University	N/A
Software and Algorithms		
Accuri CSampler	BD Biosciences	N/A
FlowJo	Treestar	N/A

(*Continued on next page*)

Continued

REAGENT or RESOURCE	SOURCE	IDENTIFIER
Volocity version 6.3	PerkinElmer	N/A
Photoshop CC	Adobe Systems	N/A
Illustrator CC	Adobe Systems	N/A
qPCR software	Biorad	N/A
Graphpad Prism	GraphPad Software	N/A
SerialEM software package	N/A	N/A
IMOD software package	N/A	N/A
Other		
Luminometer	Berthold Technologies	N/A
Accuri C6	BD Biosciences	N/A
Leica Cryostat CM1950	Leica	CM1950 (Pietro Di Camilli Lab)
Leica TCS DMi8 SP8 microscope	Leica	CCMI Yale Central Facility
HPM-010 high-pressure freezing machine	Leica Microsystems, Vienna Austria	N/A
AFS-2 freeze-substitution machine	Leica Microsystems	N/A
Stereo dissecting microscope	Nikon	SMZ645
UC6 ultramicrotome	Leica Microsystems	N/A
Transmission electron microscope	Tecnai	TF30ST-FEG
2k x 2k CCD camera	Gatan, Inc	XP1000
C1000 Touch thermal cycler	Bio-Rad	N/A
CFX Connect Real-Time PCR Detection System	Bio-Rad	N/A
Nanodrop Spectrophotometer ND-1000	Thermo Fisher Scientific	N/A
27G × ½'' insulin syringe with needle	TERUMO	Cat # SS*05M2713
31G insulin syringe	BD Biosciences	Cat # 328468
70 μm Nylon cell strainer	FALCON	Cat # 352350
Acrodisc 25 mm Syringe Filter w/0.45 μm HT Tuffryn Membrane	PALL Life Sciences	Cat # 4184
HiTrap Protein G HP antibody purification columns	GE Healthcare Life Sciences	Cat # 29048581
Superfrost Plus Microscope Slides	Thermo Scientific	Cat # 4951PLUS-001
96-well white plates for luciferase assays	Costar	Cat # 3917
Accu-Edge High Profile Microtome Blades	SAKURA	Ref # 4685
Microcover glasses 1 ounce No.1	VWR	Cat # 48393 106
Tissue-Tek Cryomold	SAKURA	Ref # 4557
Brass planchettes	Ted Pella	Type A
Brass planchettes	Ted Pella	Type B
Cryotubes	Nunc	N/A
Teflon-coated glass microscope slides	N/A	N/A
Microsurgical scalpel	N/A	N/A
Plastic sectioning stubs	N/A	N/A
Diamond knife	Diatome, Ltd	N/A
Formvar-coated copper-rhodium slot grids	Electron Microscopy Sciences	N/A
Dual-axis tomography holder	E.A. Fischione Instruments, Export PA	Model 2040
Polystyrene Round-bottom Tube	FALCON	Ref # 352058
Optical Flat 8-Cap Strips for 0.2 ml tube stripes/plates	Bio-Rad	Cat # TCS0803
Individual PCR tubes 8-tube Strip, clear	Bio-Rad	Cat # TLS0801
ThermalGrid Rigid Strip PCR tubes	Denville Scientific	Ref # C18064
96 well U bottom plate	FALCON	Ref # 353077
Easy-Sep Magnet	Stemcell	Cat # 18000

CONTACT FOR REAGENT AND RESOURCE SHARING

Further information and requests for resources and reagents should be directed to and will be fulfilled by the Lead Contact, Walther Mothes (walther.mothes@yale.edu).

EXPERIMENTAL MODEL AND SUBJECT DETAILS

Mice

C57BL/6 (B6), BALB/cJ, NagyDsRed.T3 and UBI-GFP mice were obtained from Jackson Laboratory. CD169$^{-/-}$ mice (B6 background) were from Paul Crocker, University of Dundee UK (Oetke et al., 2006). Requests for CD169$^{-/-}$ mice should be directed to Paul Crocker. *Ifnar1*$^{-/-}$ mice were from Akiko Iwasaki, Yale University. *Ifnar1*$^{-/-}$CD169$^{-/-}$ mice were generated in this work by crossing CD169$^{-/-}$ mice with *Ifnar1*$^{-/-}$ mice. B6.A-*Fv2*$^{s/s}$ mice were from the George Kassiotis of the Francis Crick Institute (former National Institute for Medical Research), UK (Antunes et al., 2008). Requests for B6.A-*Fv2*$^{s/s}$ should be directed to George Kassiotis. *Fv2*$^{s/s}$CD169$^{-/-}$ mice were generated in this work by crossing B6.A-*Fv2*$^{s/s}$ mice with CD169$^{-/-}$ mice. All the animals were housed under specific pathogen-free conditions in the facility of Yale Animal Resources Center (YARC). FrMLV env-specific TCR-transgenic mice (EF4.1 strain TCR-β transgenic mice) were generated and maintained at the Francis Crick Institute (former National Institute for Medical Research), UK. *Batf3*$^{-/-}$ mice were from Stephanie Eisenbarth, Yale University. All experiments were approved by the Institutional Animal Care and Use Committees (IACUC) of and Institutional Biosafety Committee of Yale University. 6–8 week old male and female mice were used for all the experiments. NagyDsRed.T3 and UBI-GFP mice of the same sex as the recipient mice were used in the *in vivo* CTL experiments.

Virus Production and Titration

Friend Virus Complex

A stock of Lactate dehydrogenase-elevating virus (LDV)-free FVC was used for the study. They were prepared by retro-orbital infection of BALB/cJ mice and harvesting spleens at 8 dpi. 10 % spleen homogenates were made in serum-free RPMI by passing through 75 μm mesh. Excess cells were removed by sedimentation and aliquots of supernatants were stored at -80°C. Titers of fresh virus stocks were determined in BALB/cJ mice 8 days after retro-orbital administration of diluted virus stocks by counting the foci on the spleen stained with Bouin's solution and expressed as spleen focus forming units (SFFU). An amount of virus equivalent to 2,500 SFFU or 500 SFFU as indicated was used for our experiments. In addition, serial dilutions of viruses were plated on DFJ8 cells (DF-1 chicken cells expressing FrMLV receptor mCAT-1) and cultured for 36-48 h. The cells were fixed and stained with antibodies to Glycogag (purified from culture supernatants of mAb34 hybridoma; conjugated to Alexa 647) and analyzed by FACS to estimate titers in terms of infectious units.

For cryo-histology experiments we generated FVCYpet and FVC GFP that express fluorescent proteins in the cytoplasm of infected cells. For producing FVCYpet, we first generated a full-length replication competent MLV in pLRB303 backbone expressing the fluorescent protein Ypet (FrMLVYpet) inserted after the envelope ORF under a modified IRES (6ATRI) using a strategy described earlier (Alberti et al., 2015; Logg et al., 2001; Nguyen and Daugherty, 2005; Yoon et al., 2013). FVCYpet was made by co-transfecting HEK293 cells with equal amounts of plasmid DNA encoding FrMLVYpet and HindIII fragment released from pBR322 plasmid encoding SFFV LS strain (gift from Frank Malik and Leonard Evans). For FVC GFP, a BamHI to BlpI fragment encoding the SFFV gp55 from pBR322 SFFV LS plasmid was inserted in to pLRB303 backbone digested with BamHI and BlpI. Next, a Pac I site was introduced using site directed mutagenesis right after the Gp55 ORF to insert a 6ATRI GFP cassette for obtaining the pLRB303 SFFV GFP construct. FVC GFP was made by co-transfecting HEK293 cells with equal amounts of plasmid DNA encoding FrMLV (pLRB303) and SFFV (pLRB303 SFFV GFP). Culture supernatants were harvested 48 h later and *in vitro* virus titers were determined by infecting murine T lymphoid cell line S49.1 for 24 h followed by flow cytometry to enumerate MLV glycoGag, Ypet and GFP expressing cells. FVCYpet or FVC GFP particles were concentrated by sedimentation through a 15 % sucrose-PBS cushion. Concentrated virus (equivalent to 2 x 10^6 infectious units, *in vitro*) was suspended in phosphate buffered saline (PBS) containing 0.1% bovine serum albumin (BSA) and administered s.c. into the footpad or retro-orbitally for infection of mice.

FrMLV Expressing GFP and Luciferase

FrMLV expressing GFP were generated by co-transfecting HEK293 cells with plasmids pLRB303-FrMLV (encodes full-length replication competent Friend57 MLV) (Oliff et al., 1980) and pMMP-LTR-GFP (encodes cytoplasmic GFP driven by MLV LTR) at a ratio of 10:1 (Sewald et al., 2015). FrMLV expressing luciferase was generated similarly by co-transfection with pMIGw-Antares generated in this study by replacing IRES GFP cassette with Antares luciferase from pNCS-Antares. pMIG-w was a gift from Luk Parijs (Addgene plasmid # 12282) and pNCS-Antares was a gift from Michael Lin (Addgene plasmid # 74279) (Chu et al., 2016; Refaeli et al., 2002). The culture supernatants were harvested 48 h later, filtered, aliquoted and stored at -80°C. Virus titers were determined by infecting murine T lymphoid cell line S49.1 for 24 h or DFJ8 cells for 36-48 h followed by flow cytometry to enumerate MLV Glycogag, GFP or Antares expressing cells. Antares luciferase activity was monitored where applicable in cell lysates of infected cells in 1X passive lysis buffer using 1 in 40 dilution of the Nano-Glo luciferase assay reagent (Promega Corp) and luminometer (Berthold technologies). Viral particles in the culture supernatants were concentrated by sedimentation through a 15 % sucrose-PBS cushion. Concentrated virus (equivalent to 4 x 10^5 infectious units, *in vitro*) was suspended in phosphate buffered saline (PBS) containing 0.1% bovine serum albumin (BSA) and injected with 31 guage insulin syringes either retro-orbital (r.o.) or s.c. into the footpad of mice.

For producing MLV Gag-GFP labeled virions, we generated a construct where eGFP was introduced in frame at the C-terminus of the Gag gene in the full length MLV context using pLRB303 plasmid ($FrMLV_{FL}$ Gag-GFP) as described previously (Jin et al., 2009). This renders the virus replication defective as the Pol gene is non-functional. MLV particles were generated by transfecting cells with this $FrMLV_{FL}$ Gag-GFP in the additional presence of a plasmids expressing MLV GagPol and FrMLV Env at a ratio of 6:3:1 (Jin et al., 2009). For capture experiments, we retroorbitally injected a virus amount equivalent to 2 x 10^6 infectious units as ascertained by comparison of gag signals in sedimented virus using antibodies to MLV gag p30 (R187) by western blot (Sewald et al., 2015).

METHOD DETAILS

Retrovirus Infection and Treatment Conditions

Retrovirus infection of mice was initiated by administering 500 or 2,500 SFFU equivalent of virus (see above) into the footpad (s.c.) or via retro-orbital (i.v.) injections. For CD169-blocking experiments in BALB/cJ mice, 5 μg of antibodies to CD169 (clone 3D6.112, BioLegend, San Diego, CA, USA) or rat IgG2a isotype control were injected into the footpad (s.c.) 24 h and 30 min prior to virus injection and/or every 48 h thereafter for the duration of the experiment to block CD169 at the pLN. For the $CD8^+$ T cell depletion experiment, 250 μg of $CD8^+$ T cell depletion antibody (clone YTS 169.4, Cat # BE0117, Bio X Cell) was administered intraperitoneally (i.p.) into the mice at 5 dpi (s.c) with FVC. Lymphocyte emigration from lymphoid tissues was inhibited by intra-peritoneal (i.p.) administration of FTY720 (1 μg per gram of body weight) or equivalent amount of vehicle 24 h prior to virus inoculation and every 24 h for the duration of the experiment. For survival experiments, mice were monitored every 6-12 h starting six days after virus administration. Lethargic and moribund mice were sacrificed and considered to have succumbed to infection for Kaplan-Meier survival plots.

Single Cell Preparation from Mouse Tissue

Popliteal lymph nodes and spleens harvested after necropsy were disrupted in serum free media, treated with Liberase TL (0.2 mg/ml, Sigma-Aldrich, Cat # 5401020001) and DNase I (20 μg/ml, Roche, REF # 04716728001) at 37°C for 20 min and passed through a 70 μm cell strainer (Falcon, Cat # 352350). Splenic cell suspensions were treated additionally with red blood cell lysis buffer at room temperature for 10 min (Sigma-Aldrich, Cat # R7757-100ML or BioLegend, Cat # 420301) for removing RBCs to obtain single cell suspensions. Single cells suspensions from each lymphoid tissue were stimulated *ex vivo*, cultured for functional analysis or fixed with 4 % PFA (Cat # 19200, Electron Microscopy Sciences) before processing for flow cytometric analysis.

Monitoring Virus Particle Flow

The estimation of viral load 1 h post s.c. infection at the draining popliteal lymph node, serum and spleen was carried out using FrMLV luciferase reporter virus and amplified using highly susceptible DFJ8 cells. Virus that was equivalent to 5.8×10^7 I.U. was injected s.c. into mice. Dilutions of single cell suspensions from lymph node and spleen as well as serum collected through heart-puncture was incubated with 1.25×10^4 DFJ8 cells in a 48-well plate. 36-48 h later, DFJ8 cells were lysed with 150 μl 1X passive lysis buffer (Promega Corp). 25 μl of lysate was tested for nanoluc activity using the Nano-Glo Luciferase Assay System (Cat # N1120, Promega) in a luminometer (Berthold technologies). Cell lysates from uninfected DFJ8s were used for normalization and determining relative luminescence units.

Glycogag-Alexa Conjugates

mAb34 (anti-MLV Glycogag) hybridoma (Chesebro et al., 1981) was cultured in Hybridoma-SFM media supplemented with Ultra-low Ig FBS (Cat # 16250-086, Life technologies) in 15 cm tissue culture dishes. Culture supernatants were collected every three days followed by passage through 0.45 μm low-protein binding cellulose acetate filters. Filtrate was diluted 9:1 with 200mM phosphate buffer containing 82 mM NaCl (pH 7.0) and loaded to a HiTrap Protein G HP column (GE Healthcare, USA). Column-bound antibodies were eluted with 0.1 M glycine (pH 3.0) and concentrations of purified antibodies were measured using Nanodrop Spectrophotometer ND-1000. 100 μg of antibody was conjugated to AF488A or AF647 using the Mix-n-Stain antibody labeling kit (Cat # MX488AS100 and MX647S100, Biotium, Sigma-Aldrich) and stored in antibody storage buffer at 4°C for routine use.

Flow Cytometric Analyses

PFA-fixed cells from lymph nodes and spleens were blocked for 15 min in PBS containing 2 % BSA, 5 % rat serum and Fc blocking antibody against CD16/CD32 (BioLegend) before staining with antibodies listed in the table above for flow cytometry analysis. FVC-infected erythroblasts were determined by gating for cells that were $Glycogag^+$ $CD71^+$ $Ter119^+$ in the $CD19^-$ population. Similarly double positive $CD71^+$ $Ter119^+$ were gated out to determine FVC-infected $CD19^+$ $Glycogag^+$ B cells. All staining for flow cytometry was performed in staining buffer (1X PBS containing 2% FBS, 1% BSA and 0.2% gelatin). For intracellular staining, cells are permeabilized with staining buffer supplemented with 0.2 % Triton X-100. The details of the antibodies used in the study are listed in key sources table. Data were acquired on an Accuri C6 (BD Biosciences) and were analyzed with Accuri C6 or FlowJo software (Treestar). 200,000 – 500,000 viable cells were acquired for each sample. Each data point represents results from a single lymph node or spleen as indicated.

DFJ8 Co-culture for Determining Infectivity

We employed DFJ8 co-culture assay for enhancing our sensitivity to determine productively infected cells in pLN and spleen. 5×10^5 cells from each pLN or 2×10^6 splenocytes were co-cultured with 1×10^5 DFJ8 cells in 24-well plate for 48 h. The co-cultured cells were washed thrice with 1X PBS to remove all the non-adhered cells, treated with 0.05 % trypsin and fixed with 4 % PFA. Infected DFJ8 cells gated by FACS as Glycogag$^+$ CD45.2$^-$ cells were used to determine the level of infection.

Cryo-Immunohistology of Spleen

FVC-infected spleens were harvested 5 dpi (2,500 SFFU, r.o.) and fixed in 1X PBS containing freshly prepared 4 % PFA for 12 h at 4°C. The spleens were washed with PBS, cryoprotected with 10, 20 and 30 % ascending sucrose series, snap-frozen in Tissue-Tek O.C.T. compound and stored at –80°C. 15-30 μm thick sections were permeabilized with Triton X-100 and treated with Fc receptor blocker (Innovex Biosciences) before staining with indicated antibodies in PBS containing 2 % BSA. Stained sections cured with Pro-Long Gold antifade reagent were analyzed by confocal microscopy using Leica TCS SP8 microscope equipped with white light laser. The images were processed using Volocity version 6.3 software (PerkinElmer, Waltham, MA, USA) and figures assembled with Photoshop CC and Illustrator CC (Adobe Systems, San Jose, CA, USA).

Sample Preparation for Electron Microscopy

Spleens from B6 and CD169$^{-/-}$ mice were challenged retro-orbitally with FVC (2,500 SFFU, 5 dpi) isolated, divided into 8 equal pieces and immediately fixed with 3 % glutaraldehyde, 1 % paraformaldehyde, 5 % sucrose in 0.1 M sodium cacodylate trihydrate. Pre-fixed pieces of spleen were rinsed with fresh cacodylate buffer and placed individually into brass planchettes (Type A; Ted Pella, Redding, CA) prefilled with 10 % Ficoll in cacodylate buffer. The tissues were covered with the flat side of a Type-B brass planchette and rapidly frozen with a HPM-010 high-pressure freezing machine (Leica Microsystems, Vienna Austria). The frozen samples were transferred under liquid nitrogen to cryotubes (Nunc) containing a frozen solution of 2.5 % osmium tetroxide, 0.05 % uranyl acetate in acetone. Tubes were loaded into an AFS-2 freeze-substitution machine (Leica Microsystems) and processed at -90°C for 72 h, warmed over 12 h to -20°C, held at that temperature for 6 h, then warmed to 4°C for 2 h. The fixative was removed and the samples rinsed 4 x with cold acetone, following which they were infiltrated with Epon-Araldite resin (Electron Microscopy Sciences, Port Washington PA) over 48 h. The spleen tissue was flat-embedded between two Teflon-coated glass microscope slides. Resin was polymerized at 60°C for 48 h.

Electron Microscopy and Dual-Axis Tomography

Flat-embedded splenic samples were observed with a stereo dissecting microscope and appropriate regions were extracted with a microsurgical scalpel and glued to the tips of plastic sectioning stubs. Semi-thick (400 nm) serial sections were cut with a UC6 ultramicrotome (Leica Microsystems) using a diamond knife (Diatome, Ltd. Switzerland). Sections were placed on formvar-coated copper-rhodium slot grids (Electron Microscopy Sciences) and stained with 3 % uranyl acetate and lead citrate. Gold beads (10 nm) were placed on both surfaces of the grid to serve as fiducial markers for subsequent image alignment. Sections were placed in a dual-axis tomography holder (Model 2040, E.A. Fischione Instruments, Export PA) and imaged with a Tecnai TF30ST-FEG transmission electron microscope (300 KeV) equipped with a 2k x 2k CCD camera (XP1000; Gatan, Pleasanton CA). Tomographic tilt-series and large-area montaged overviews were acquired automatically using the SerialEM software package (35). For tomography, samples were tilted +/- 64° and images collected at 1° intervals. The grid was then rotated 90° and a similar series taken about the orthogonal axis. Tomographic data was calculated, analyzed and modeled using the IMOD software package (36, 37) on MacPro computers (Apple, Cupertino, CA). Lower resolution montaged overviews were used to identify cell types and frequency within the tissue sections. High-resolution electron tomography was used to confirm and characterize virus particles and budding profiles on the surfaces of infected cells.

Neutralizing Antibody Titer

Mice were bled retro-orbitally at days 7, 14, and 21 post infection with FVC (2,500 SFFU) and allowed to clot at room temperature for 1 h. The samples were sedimented at 14,000 rpm for 30 min at 25°C to collect the sera and stored at -80°C. Serial two-fold dilutions of indicated heat-inactivated (56°C for 30 min) serum samples were incubated for 1 h at 37°C with MLV expressing Antares luciferase (luciferase values corresponding to 2×10^4 I.U.) and 1 μl of guinea pig complement (MP Biomedical,1:64 hemolytic titer) in a total volume of 50 μl (serum-free media) in a 96-well plate. Equal volumes of media with 2X serum containing S49.1 T cells (2×10^5) were added to each well and incubated further at 37°C for 24 h. Sedimented cells were lysed using 1X passive lysis buffer (Promega Corp) and the luciferase activity was measured as above. Luciferase activity in samples with pooled sera from uninfected mice was set as 100 %. The log (dose-dependent inhibition/ sera dilution) was plotted against log of sera dilution to fit a linear regression. The slope was used to calculate IC_{50} values defined as the amount of serum that neutralizes half the MLV infectivity. The IC_{50} values were analyzed for statistical significance by applying correction for multiple comparisons using the Bonferroni-Dunn method using multiple t-test option in GraphPad Prism v6.0.

FVC-Specific CD4$^+$ T Cell Proliferation

Previous studies have shown that FVC-specific CD4$^+$ T cells constitute a very small proportion of the entire the CD4$^+$ T cell population precluding direct assessment of their proliferation potential (Antunes et al., 2008). Therefore, we used EF4.1 mice, which

transgenically express a TCRβ chain that can specifically recognize MHC class II-presented FrMLV envelope epitope to compare FVC-specific $CD4^+$ T cell responses in B6 and $CD169^{-/-}$ mice (Antunes et al., 2008). $CD45.1^+$ $CD4^+$ T cells were isolated from spleens of EF4.1 mice using a negative selection $CD4^+$ T cell enrichment kit (Cat # 19752A, STEMCELL Technologies). 1×10^6 cells were adoptively transferred (r.o) with 27 guage insulin syringes into either CD45.2 uninfected or FVC-infected B6 or $CD169^{-/-}$ mice 8 dpi (s.c, 2500 SFFU). Recipient mice were sacrificed 4 days post transfer and cells from pLNs and spleens analyzed for proliferating $CD44^{hi}$ antigen-experienced adoptively transferred $CD45.1^+$ $CD4^+$ T cells by FACS.

Type I Interferon Response

Mice were challenged s.c. (2,500 SFFU) for 8 hr or r.o. (2500 SFFU) for 12 h with FVC. RNA was extracted from single cell suspensions of an entire popliteal lymph node or from 1×10^7 splenocytes with RNeasy Mini Kit (QIAGEN Catalog number 74104). Contaminating DNA was removed with DNase I (Cat # AM2222, Ambion, Thermo Fisher Scientific) followed by a treatment with a DNase I inactivation reagent (Cat # 8173G, Ambion). 100 ng of RNA from each sample was used for cDNA synthesis with qScript cDNA Synthesis kit (Quanta Biosciences, Cat # 95047-100). cDNAs were used for quantitative PCR analyses to determine the mRNA levels of type I interferon (IFN) and interferon-stimulated genes (ISGs) with a SYBR FAST qPCR Master Mix (2X) Kit (KAPA Biosystems, Cat # KK4600 and KK4601). CFX Connect Real-Time PCR Detection System (Bio-Rad Laboratories) was used for carrying out quantitative PCR and the data analyzed using the built-in CFX Maestro Software. The primers used for amplification are as listed above. The PCR conditions were 95°C 3 min, 40 cycles of 95°C for 15 s and 60°C for 1min, followed by a melting curve analysis to ensure that each primer pair resulted in amplification of a single PCR product. mRNA levels of *IFNB1*, *IFNA2* and *IFNA4* in the cDNA samples of infected mice were normalized to actin with the formula $2^{-[Ct\ (IFN)-Ct(actin)]}$. The fold increase in the mRNA level of ISGs in an infected mouse compared to the average in three uninfected mice was calculated as $2^{-\{[Ct(ISG\ infected)-Ct(actin\ infected)]-average\ of\ [Ct(ISG\ noninfected)-Ct(actin\ noninfected)]\}}$ (Schmittgen and Livak, 2008).

Measuring CTL Activity *In Vivo* and *In Vitro*

Single cell suspensions of splenocytes from a NagyDsRed.T3 and a UBI-GFP mouse were prepared as described above. 1×10^8 cells / ml suspensions of NagyDsRed.T3 and UBI-GFP splenocytes were incubated with 6 μM FrMLV Gag-specific peptide (KKCCLCLTVFL) or same amount DMSO respectively for 2 h at 37°C. For measuring *in vivo* CTL activity, A 1:1 ratio (2 $\times10^6$ cells of each population) was injected retro-orbitally (r.o.) using a 27-guage needle for adoptive transfer into uninfected or FVC-infected mice (7 dpi ; r.o.). The recipient mice were euthanized 24 h later and FVC-specific CTL activity analyzed in harvested pLNs and spleens by estimating the ratio of $DsRed.T3^+$ and GFP^+ cells by FACS. We acquired a minimum of 7000-5000 adoptively transferred non-pulsed GFP^+ cells for analyses.

For measuring CTL activity, *in vitro*, effector $CD8^+$ T cells were purified from the spleens of non-infected mice or FVC-infected mice (7 dpi; r.o.) using a negative selection-based $CD8^+$ T cell enrichment kit (STEMCELL Technologies). 1.5×10^5 cells comprising 1:1 ratio of peptide-pulsed $DsRed.T3^+$ and non-pulsed GFP^+ splenocytes prepared as above were used as targets. Effector $CD8^+$ T cells were added to target cells at a ratio of 1:1, 2:1, 4:1 and 8:1 and co-cultured in a U-bottom of 96-well plate for 24-36 h. Cells were fixed and ratios of $DsRed.T3^+$ and GFP^+ cells analyzed by FACS as above.

Specific CTL activity was calculated as below according the formula described by Quah et al. (2012)

$$\%\ Specific\ killing = \left[1-\left(\frac{Targets^{DsRed}_{infected}/Targets^{GFP}_{infected}}{Targets^{DsRed}_{noninfected}/Targets^{GFP}_{noninfected}}\right)\right]\times 100$$

$CD8^+$ T Cell Analyses

In vitro culture and stimulation of cells were performed in U-bottom 96-well plates with 1X RPMI 1640 medium supplemented with 10% FBS, 1X MEM Non-essential amino acid (NEAA) solution, 1mM sodium pyruvate and 56 μM β-mercaptoethanol. CD107A staining to determine degranulation activity of FVC-specific $CD8^+$ T cells was carried out by stimulating 2×10^6 splenocytes from uninfected or FVC-infected mice (7-8 dpi) with 6 μM FrMLV Gag-specific peptide and 1:500 diluted AF647 anti-mouse CD107a (clone 1D4B). GolgiStop (BD Biosciences, San Jose, CA, USA) was added 1 h later and the splenocytes cultured for additional 12-15 h. Cells were fixed and stained with antibodies to CD3 and CD8 to identify $CD107A^+$ $CD8^+$ cells in the $CD3^+$ T cell population. For intracellular IFNγ staining, 2×10^6 splenocytes from uninfected or infected mice were re-stimulated *in vitro* with peptide as above for 16 h in presence of 2 μg/ml Brefeldin A to inhibit secretion of IFNγ. In addition, we also monitored IFNγ in single cell suspensions from lymph nodes (1 x 10^6 cells) or spleen (2 x 10^6 cells) of infected mice after generalized stimulation with 50 ng/mL PMA and 1 μM ionomycin for 3 h in RPMI containing GolgiStop. Granzyme A and B staining was carried out on splenocytes stimulated with peptide 16-24 h without GolgiStop.

$CD8^+$ T Cell Adoptive Transfer

Single cell suspensions of splenocytes from FVC-infected B6 and $CD169^{-/-}$ mice (7 dpi; r.o. 2,500 SFFU) were prepared as described above. Effector $CD8^+$ T cells were purified from the splenocytes using a negative selection-based $CD8^+$ T cell enrichment kit (STEMCELL Technologies). 6.4×10^6 $CD8^+$ T cells from infected B6 or $CD169^{-/-}$ mice were injected retro-orbitally (r.o.)

using a 27-gauge needle for adoptive transfer into infected CD169$^{-/-}$ mice (5 dpi; r.o. 2,500 SFFU). The recipient mice were euthanized 2 days later and infected cells were analyzed in harvested spleens.

QUANTIFICATION AND STATISTICAL ANALYSIS

Statistical comparisons were performed using non-parametric Mann-Whitney test (two-tailed) available in GraphPad Prism software (La Jolla, CA, USA). Statistical analyses for multiple comparisons such as shown in Figure S4H was calculated by applying correction for multiple comparisons using the Bonferroni-Dunn method using multiple t-test option in GraphPad Prism v6.0. Exact P values and the numbers of independent replicates (n) are mentioned in the figures or figure legends. A difference was considered significant if $P < 0.05$.

Cell Host & Microbe, Volume 25

Supplemental Information

A Protective Role for the Lectin CD169/Siglec-1 against a Pathogenic Murine Retrovirus

Pradeep D. Uchil, Ruoxi Pi, Kelsey A. Haugh, Mark S. Ladinsky, John D. Ventura, Brad S. Barrett, Mario L. Santiago, Pamela J. Bjorkman, George Kassiotis, Xaver Sewald, and Walther Mothes

Supplemental Information:

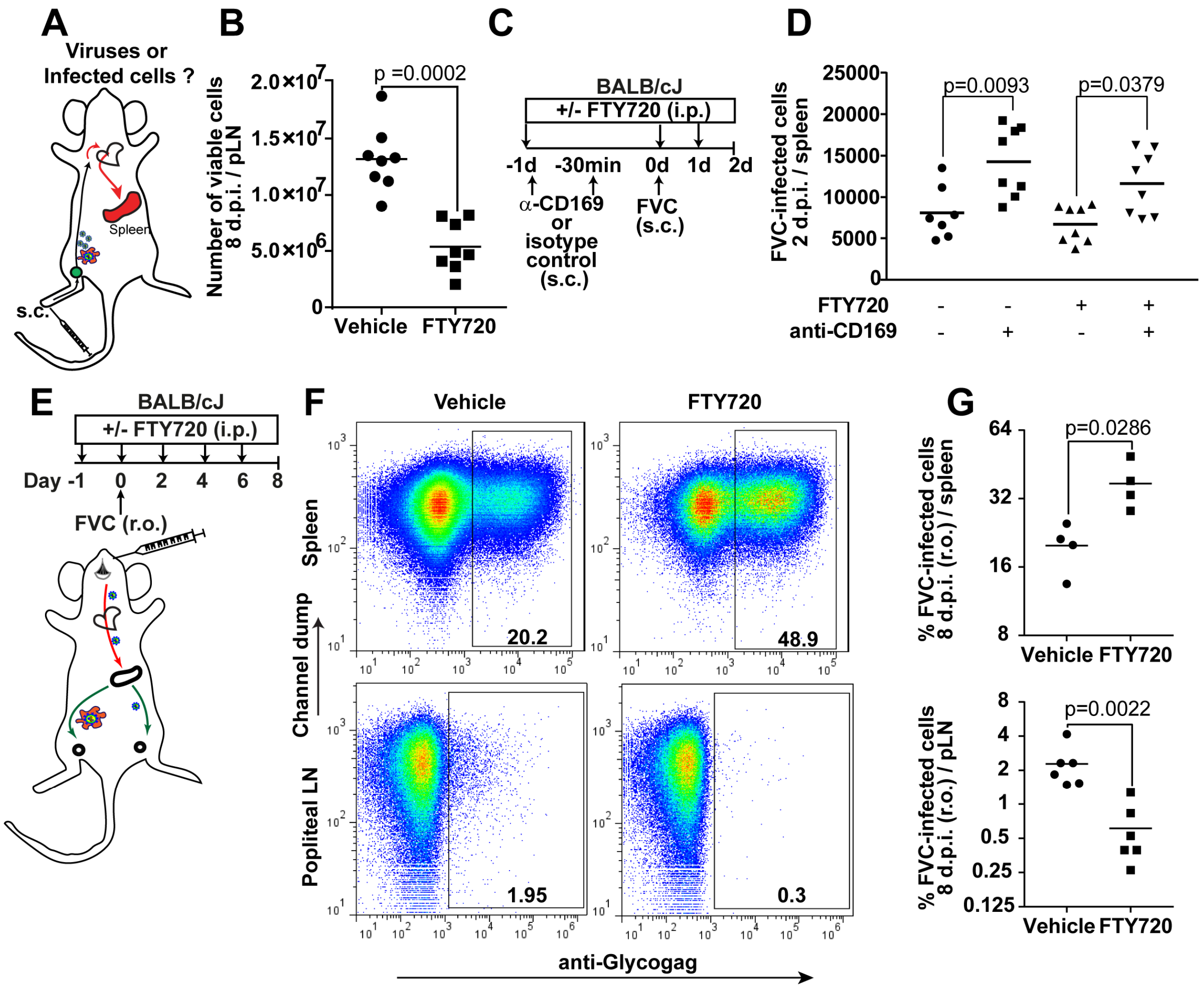

Figure S1, Related to Figure 2. Retroviruses Use Both Cell-free and Cell-Associated Modes for Dissemination in Mice

(A) Scheme depicting possible spread of infection via cell-free viruses (blue) or virus-infected cells (orange) from the draining pLN via the subclavian vein (break in the arrow) to the spleen following s.c. administration of virus.

(B) An experiment to show block in lymphocyte infiltration upon FTY720 treatment. After infection, the primary draining lymph node allows the normal entry of lymphocytes from circulation to sample and identify antigen-specific lymphocytes that then proliferate leading to lymph node expansion. FTY720 treatment blocks egress of lymphocytes from all lymphoid

tissue. Thus, fewer lymphocytes enter the circulation as well as infected primary draining lymph node. The graph shows the number of cells in the pLN 8 days after s.c. administration of 2,500 SFFU FVC in BALB/cJ mice (n=8) treated 24 h before and every 24 h for 7 days with vehicle or FTY720. Upon FTY720 treatment the number of cells in the primary draining lymph node did not expand as normal showing efficient block in lymphocyte trafficking.

(C) Flow chart showing route and administration regimen for FTY720, antibodies to CD169 as well as virus.

(D) Number of infected cells in the spleen (n = 7-8) 2 days after s.c. administration of 2,500 SFFU FVC in mice treated with or without antibodies to CD169 or FTY720 as indicated for an experiment shown in C.

(E) Scheme depicting possible modes of spread from the spleen to the pLN after r.o. challenge via cell-free viruses (blue) or virus-infected cells (orange) The flow chart shows the route and administration regimen for FTY720 (i.p.) and FVC in BALB/cJ mice (r.o.; 500 SFFU).

(F) Representative FACS plot showing percentages of FVC-infected (Glycogag^{+}) cells after gating on live cells in the spleen and pLN of BALB/cJ mice for an experiment as in E. FTY720 treatment blocks lymphocyte egress leading to their accumulation in the spleen compared to vehicle. There is a concomitant reduction in spread of infection from the spleen to the pLN

(G) Graph showing percentages of FVC-infected cells in the spleen and pLN for an experiment as in E.

Significance p values were obtained using non-parametric Mann-Whitney statistical test.

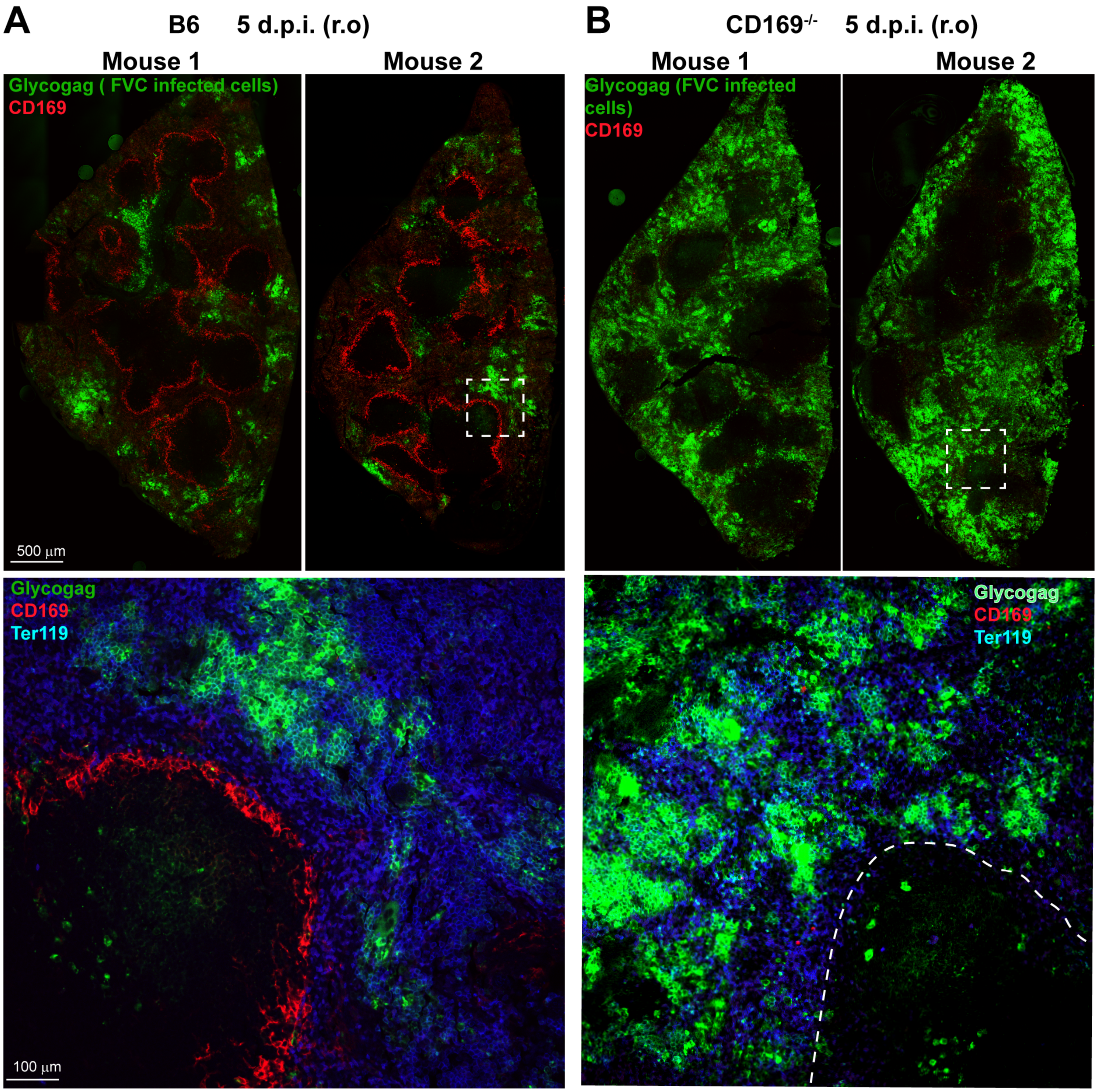

Figure S2, Related to Figure 4. FVC-infection Spreads Extensively Into the Splenic Red Pulp in CD169$^{-/-}$ Mice

(A, B) Merged stitched images of immunostained splenic tissue sections are shown for two FVC-infected B6 in A and CD169$^{-/-}$ mice in B (5 d.p.i., r.o., 2,500 SFFU). Metallophilic macrophages within white pulp and FVC-infected cells were identified using antibodies to surface marker CD169 (red) and viral protein Glycogag (green). Magnified images of indicated

insets are shown below that were labeled additionally with antibodies to Ter119 (blue) for staining cells of erythroblast lineages in the red pulp. The red and white pulp border is marked with dotted lines for clarity in B. Scale bars as indicated.

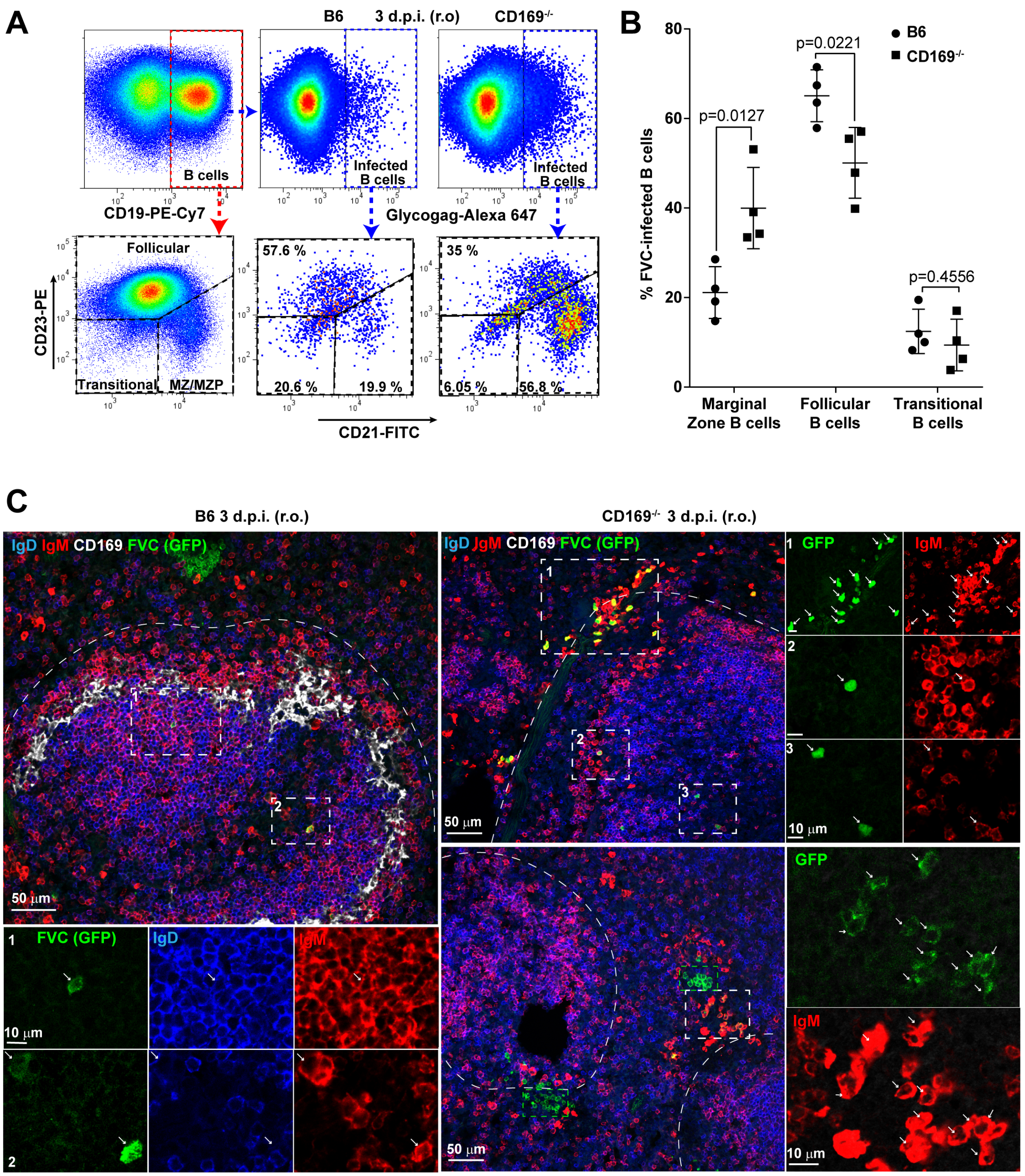

Figure S3, Related to Figure 4. CD169 Influences the Type of FVC-infected B Cells in the Splenic Marginal Zone

(A) Gating strategy and representative FACS plot showing characterization of FVC-infected (Glycogag⁺) cells in the $CD19^{+}$ B cell population for presence of mentioned markers in B6 and

CD169$^{-/-}$ mice 3 d.p.i. (n=4, s.c, 2,500 SFFU). The gating strategy used for delineating marginal zone precursor/ marginal zone B (MZ/MZP) cells from follicular (FO) and transitional B cells in the CD23 vs CD21 plot.

(B) Graph showing percentages of MZ/MZP, FO and transitional B cells in the FVC-infected B cell population for experiment as in A.

(C) Merged immunostaining images of splenic tissue sections from B6 and CD169$^{-/-}$ mice infected with FVC-expressing cytoplasmic GFP (3 d.p.i, r.o.;2 x 10^6 I.U.). MZ B cells (IgM^{hi}), FO B cells (IgD$^+$ IgM^{lo}) and metallophilic macrophages were identified using antibodies to surface markers IgM (red), IgD (blue), CD169 (white) respectively. Magnified images of individual channels from numerically labeled areas within the inset are shown either below for B6 or towards the right for CD169$^{-/-}$ mice. GFP$^+$ FVC-infected B cells types (FO and MZ) within the follicle in B6 mice are indicated with arrows. Examples of infected B cells in splenic sections of CD169$^{-/-}$ mice that are largely IgM^{hi} near the marginal zone (dotted white lines) as well as in white pulp are also shown with arrows. Green boxes demarcate clusters of infected erythroblasts that are close to the marginal zones and IgM^{hi} MZ B cells. Scale bars as indicated.

p values; non-parametric Mann-Whitney test.

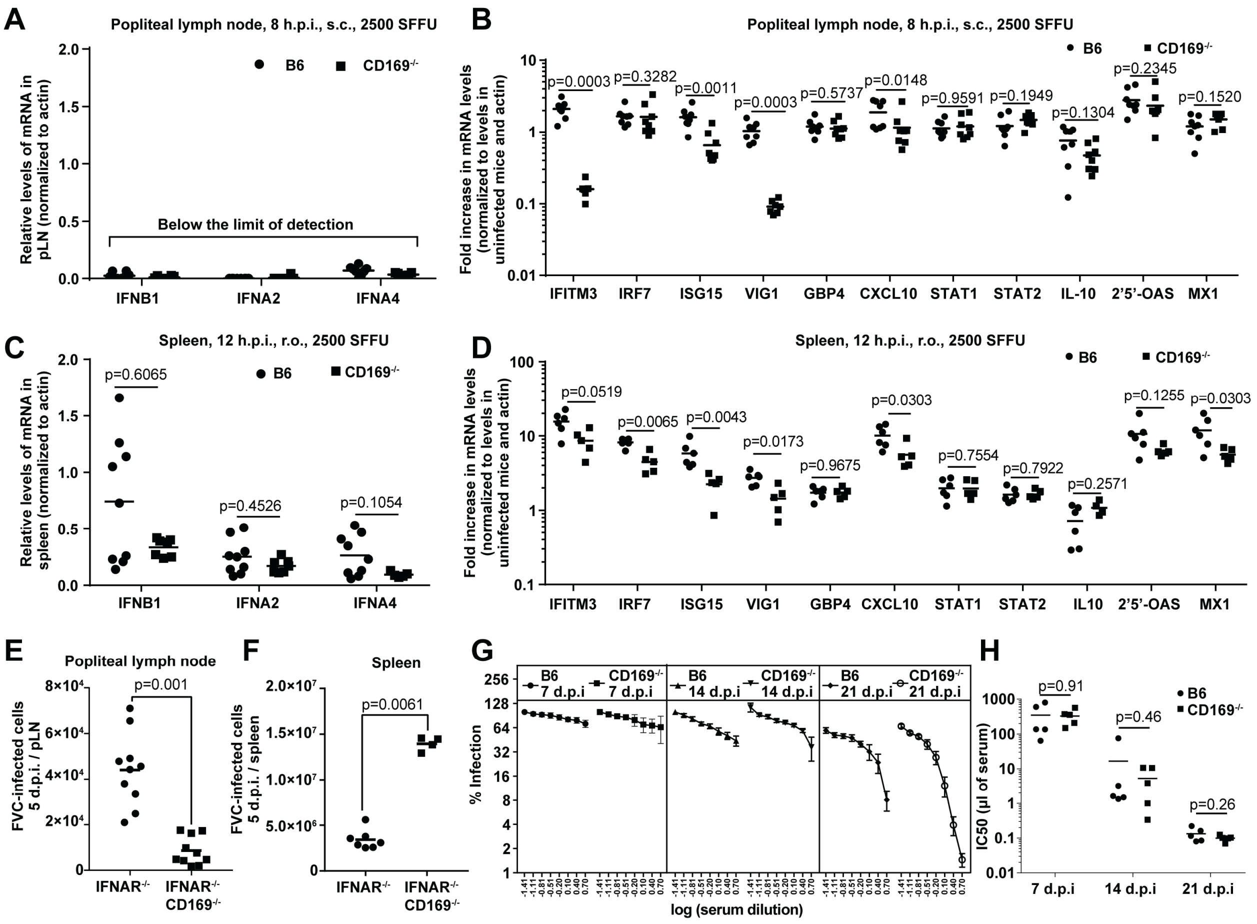

Figure S4, Related to Figure 6. Innate Immune Response and Neutralizing Antibodies Do Not Contribute to Enhanced Viral Loads in the Spleen of CD169$^{-/-}$ Mice

(A-D) mRNA levels of indicated interferons (A, C) and interferon-induced genes (B, D) in pLNs (n=6-8) and spleens (n= 4-6) of B6 or CD169$^{-/-}$ mice at specified times and indicated routes of challenge (s.c or r.o) with FVC (2,500 SFFU) as determined by real-time PCR. The mRNA levels were normalized to either actin (A, C) or actin followed by levels in uninfected cells for comparing fold increase (B, D). The levels of interferon RNA were below detection in pLNs under our experimental conditions.

(E, F) FVC-infected cells in the pLN (n = 10) and the spleen (n = 6), 5 d.p.i. after s.c. inoculation with 2,500 SFFU of FVC in *Ifnar1*$^{-/-}$ and *Ifnar1*$^{-/-}$CD169$^{-/-}$ mice.

(G) FVC-neutralizing activity in serially diluted sera from B6 and CD169$^{-/-}$ mice (n=5) at indicated times post subcutaneous challenge with FVC (2,500 SFFU).

(H) Neutralizing titers (IC_{50}) in sera from experiment shown in G.

Significance p values were obtained using non-parametric Mann-Whitney statistical test and multiple comparisons were carried out by the Bonferroni-Dunn method using multiple t-test option.

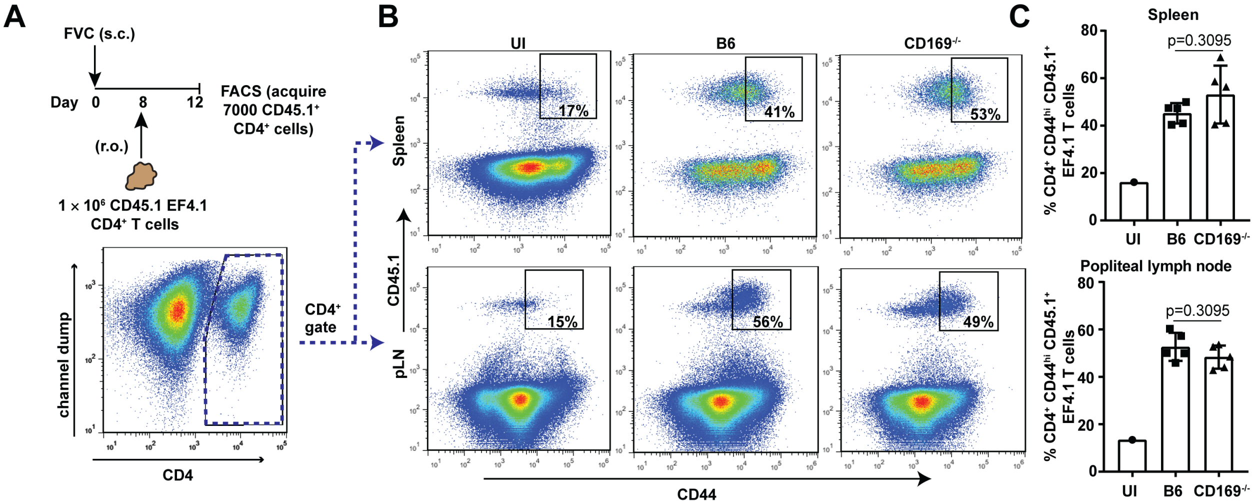

Figure S5, Related to Figure 6. FVC-Specific CD4^{+} T Cell Proliferative Responses Are Unaffected in CD169$^{-/-}$ Mice

(A) Flow chart showing experimental strategy to determine CD4^{+} T cell proliferation after adoptive transfer of CD45.1 FVC-specific EF4.1 cells in uninfected or FVC-infected B6 or CD169$^{-/-}$ CD45.2 mice (n=5).

(B) Representative FACS plots of splenocytes showing the percentage of CD44^{hi} (proliferating) cells (boxed) among the adoptively transferred CD45.1 EF4.1 cells.

(C) Graph showing percentage of CD44^{hi} proliferating EF4.1 cells at pLN and spleen for experiment shown in B.

Significance p values were obtained using non-parametric Mann-Whitney statistical test.

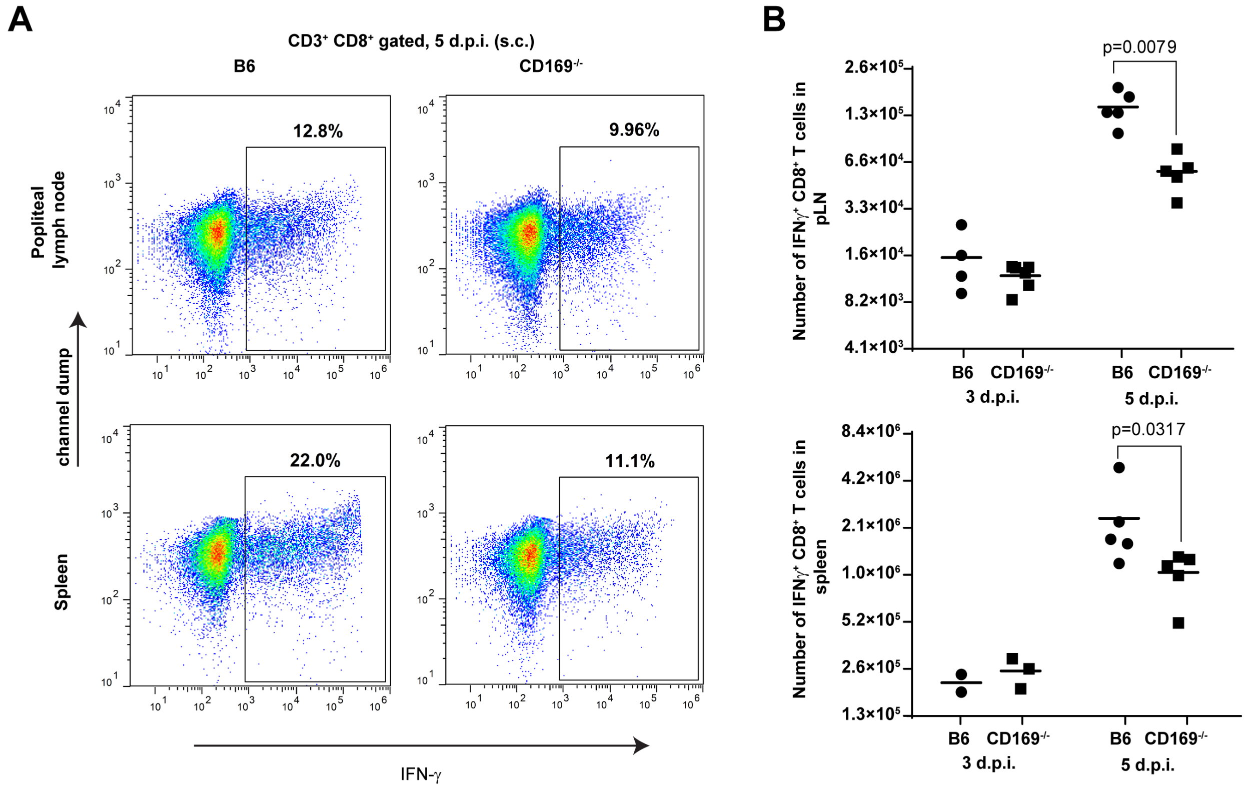

Figure S6, Related to Figure 6. Induction of IFNγ Producing CD8$^+$ T Cells is Compromised During FVC-Infection in Absence of CD169

(A) Representative FACS plot for IFNγ$^+$ cells in the CD3$^+$CD8$^+$ gated T cell population at 5 d.p.i. after s.c. delivery of 2,500 SFFU FVC in the draining pLNs (top) and spleens (bottom) from B6 and CD169$^{-/-}$ mice (n=2-5).

(B) Number of IFNγ–producing CD3$^+$CD8$^+$ T cells 3 and 5 d.p.i. for experiment as in A.

Significance p values were obtained using non-parametric Mann-Whitney statistical test.

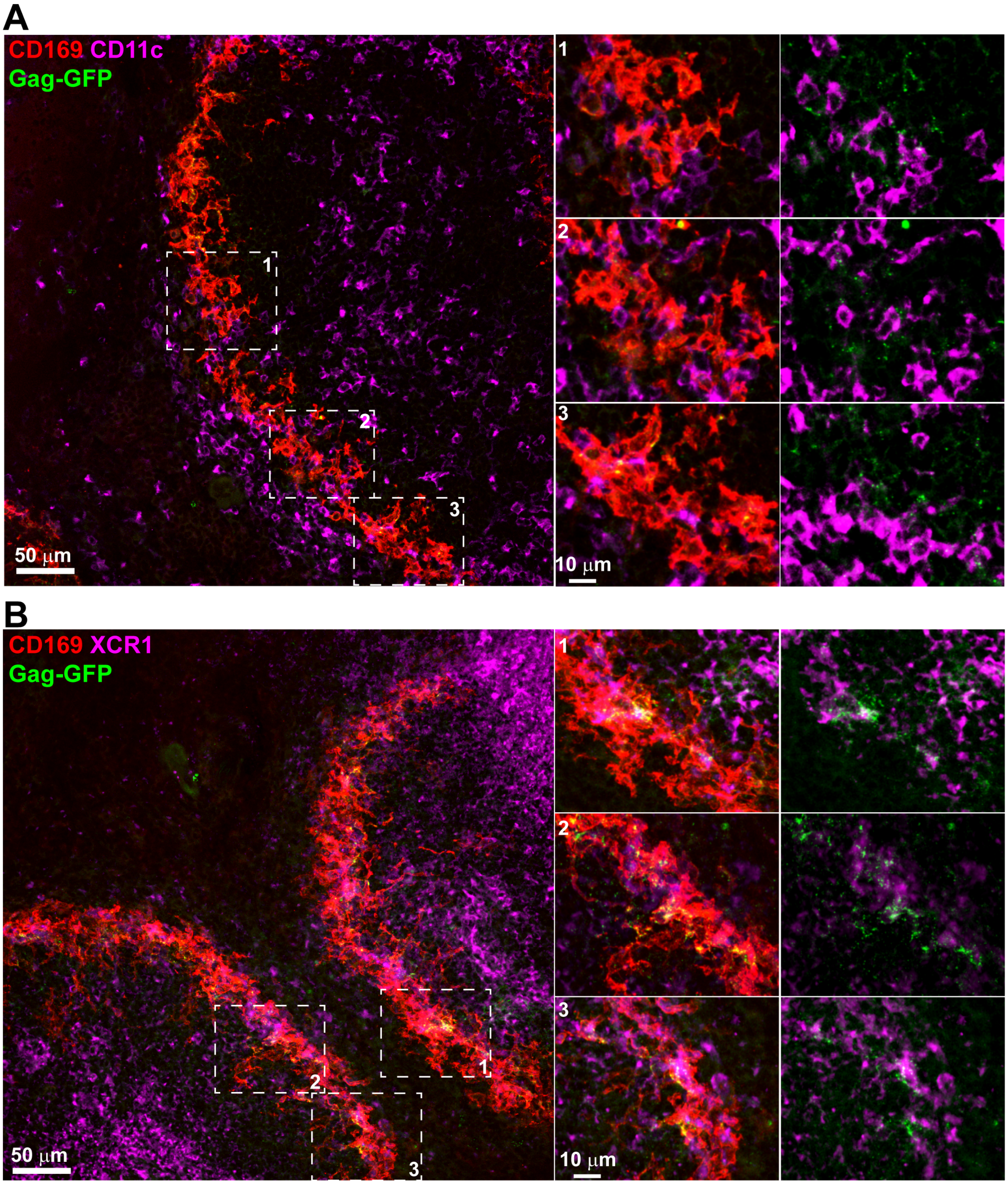

Figure S7, Related to Figure 7 and Video S2. CD11c$^+$ and XCR1$^+$ DCs Localize in Close Proximity to Retrovirus-Capturing CD169$^+$ Marginal Metallophilic Macrophages

(A, B) Merged immunostaining images of splenic tissue sections from B6 mice 2 h after r.o. administration (4 x 10^6 I.U.) of retroviral particles labeled with Gag-GFP (green). DCs, cDC1s and metallophilic macrophages were identified using antibodies to surface markers CD11c (magenta), XCR1 (magenta) and CD169 (red) respectively. Magnified images of numerically labeled areas within the inset are shown towards the right demonstrate close contacts between Gag-GFP labeled virion-capturing $CD169^+$ macrophages and $CD11c^+$ DCs (A) or $XCR1^+$ cDC1s (B).
